# Who should be prioritized for renal transplantation?: Analysis of key stakeholder preferences using discrete choice experiments

**DOI:** 10.1186/1471-2369-13-152

**Published:** 2012-11-22

**Authors:** Michael D Clark, Dennis Leech, Anil Gumber, Domenico Moro, Ala Szczepura, Nick West, Robert Higgins

**Affiliations:** 1 Warwick Medical School, University of Warwick, CV4 7AL, Coventry, UK; 2 Department of Economics, University of Warwick, Coventry, UK; 3 Centre for Health and Social Care Research, Faculty of Health and Wellbeing, Sheffield Hallam University Collegiate Campus, 32 Colliegiate Crescent, Room 205, S10 2BP, Sheffield, UK; 4 Third Sector Research Centre, University of Birmingham, Park House, 40, Egbaston Road, B15 2RT, Birmingham, UK; 5 Warwick Medical School, University of Warwick, Coventry, UK; 6 Nephrology Department, University Hospital, CV2 2DX, Walsgrave, Coventry, UK

**Keywords:** Renal transplant, Allocation, Choice experiment, Stakeholder

## Abstract

**Background:**

Policies for allocating deceased donor kidneys have recently shifted from allocation based on Human Leucocyte Antigen (HLA) tissue matching in the UK and USA. Newer allocation algorithms incorporate waiting time as a primary factor, and in the UK, young adults are also favoured. However, there is little contemporary UK research on the views of stakeholders in the transplant process to inform future allocation policy. This research project aimed to address this issue.

**Methods:**

Discrete Choice Experiment (DCE) questionnaires were used to establish priorities for kidney transplantation among different stakeholder groups in the UK. Questionnaires were targeted at patients, carers, donors / relatives of deceased donors, and healthcare professionals. Attributes considered included: waiting time; donor-recipient HLA match; whether a recipient had dependents; diseases affecting life expectancy; and diseases affecting quality of life.

**Results:**

Responses were obtained from 908 patients (including 98 ethnic minorities); 41 carers; 48 donors / relatives of deceased donors; and 113 healthcare professionals. The patient group demonstrated statistically different preferences for every attribute (i.e. significantly different from zero) so implying that changes in given attributes affected preferences, except when prioritizing those with no rather than moderate diseases affecting quality of life. The attributes valued highly related to waiting time, tissue match, prioritizing those with dependents, and prioritizing those with moderate rather than severe diseases affecting life expectancy. Some preferences differed between healthcare professionals and patients, and ethnic minority and non-ethnic minority patients. Only non-ethnic minority patients and healthcare professionals clearly prioritized those with better tissue matches.

**Conclusions:**

Our econometric results are broadly supportive of the 2006 shift in UK transplant policy which emphasized prioritizing the young and long waiters. However, our findings suggest the need for a further review in the light of observed differences in preferences amongst ethnic minorities, and also because those with dependents may be a further priority.

## Background

In the United Kingdom (UK) in January 2011 there were 6,610 patients awaiting renal transplantation (a figure which had risen by 8% annually since 2004). In the previous year (2009–10), only 1,482 patients received deceased donor transplants, and 1,038 received live donor transplants [1]. A growing imbalance between demand and supply led to the Organ Donation Taskforce Report in 2008 [2] outlining strategies to increase UK organ supply by 50% within 5 years. However, despite the resultant increase in organ supply, demand still continues to outstrip supply. So criteria remain necessary to allocate the limited supply of kidneys which are available for transplantation.

A transplant policy based on efficiency criteria would require that organs be transplanted to patients deriving greatest health benefit. Criteria to address equity of access may conflict with efficiency ones. Patients waiting a long time may be selected for transplant on equity grounds even if someone else, who has not waited as long, would obtain greater health benefits from transplantation. In 2006, UK transplant policy was re-appraised. The previous policy was thought to disadvantage those with less common tissue types and blood groups, especially ethnic minorities. This population is also at higher risk of kidney disease [3]. African Caribbeans and Asians have a 3–4 times greater risk of end stage renal disease [2] related to a higher prevalence of type 2 diabetes [4]. Increased risk of renal disease in these groups is also associated with increased risk of co-morbidities such as hypertension [5] and cardiovascular disease [6]. Moreover, ethnic minorities donate fewer organs [6], so individual patients are less likely to obtain closely matched transplants.

The 2006 re-appraisal led to reduced priority being attached to HLA matching in the UK, and allowed consideration of other criteria [7]. The resulting guidelines [8] suggested more priority should be given to long waiters and paediatric and younger adult recipients. Research from the USA and Australia had, indicated such changes would be acceptable to professionals and patients [9,10]. Although there was some excellent UK research to inform prioritization [11], this research did not adopt DCE methodology, unlike ours.

In this analysis we have used Discrete Choice Experiments (DCEs) in order to establish respondent’s valuation of different kidney transplant allocation criteria, and how they might trade-off gains in relation to one transplant allocation criterion, for losses in relation to another transplant allocation criterion. DCEs involve the application of a stated preference technique in order to establish a respondent’s valuation of attributes or characteristics of a good or service or health state. DCEs are increasingly being used to address priority setting issues in healthcare, both in primary care [12], and secondary care [13,14]. Some DCE research has been published on general transplantation issues, including assessing factors influencing willingness to donate body parts [15] and a DCE to establish UK priorities for liver transplantation [16,17]. In renal transplantation, the first DCE findings internationally emanated from our study conducted in the UK [18]. This publication focused solely on assessing whether patient preferences varied by ethnicity and gender. More recently, DCE research has been undertaken in Canada relating to patient and healthcare professional preferences for chronic kidney disease (CKD) care more generally (although not specifically focused on kidney transplantation) [19].

The current paper provides more extensive evidence on the preferences of various stakeholder groups alongside those of patients than our earlier paper [18]. These groups include patients, renal healthcare professionals, renal carers, and live donors / relatives of deceased donors. Unlike the general public (who may lack personal experience of renal disease) all these ‘expert’ stakeholder groups will have a direct interest in priorities for kidney transplant allocation, either because they have renal disease themselves (patients) or care for those with such a disease (renal carers/renal healthcare professionals). Moreover, live donors or relatives of deceased donors are concerned to ensure kidneys are appropriately allocated. Therefore research to improve understanding of the preferences of these different stakeholder groups should help inform the policy debate about transplantation.

## Methods

### Overview

This Discrete Choice Experiment (DCE) study assumed that respondents’ valuations of different kidney transplant allocation criteria can be decomposed into component parts known as attributes. The DCE involved respondents making repeated hypothetical pairwise choices in which they expressed their stated preferences about which of two transplant recipients (differing in these pre-defined characteristics or attributes) should receive a kidney. DCE respondents’ trade-offs were established so that weightings given to different recipient characteristics (attributes) could be quantified. The pilot study for this research began in 2005; the main study started in 2006 with final data analysis completed during 2007–09. Ethical approval was obtained from Warwickshire Local Research Ethics Committee (reference number 05/Q2803/86).

#### Pilot exercise

Although some DCEs have been undertaken without undertaking a pilot exercise first, the benefits of initial piloting and analysis of pilot data econometrically are considerable. We therefore undertook a rigorous pilot exercise using both qualitative and quantitative methods. For full details see Additional file 1. Attributes tested in the pilot included waiting time, tissue match, employment status, number of dependents, recipient age and various diseases affecting recipient health. Attributes and level selection was mainly informed by discussions with clinicians. Given the number of attributes and levels selected, we needed to design a DCE questionnaire which used a limited number of different choice scenarios, which would be sufficient to infer choice information. However, we also invited pilot respondents to suggest other possible attributes and rank them in order of priority (alongside those already in the questionnaire). Attributes tested in the pilot included waiting time, tissue match, employment status, number of dependents, recipient age, patient compliance, whether illness could be regarded as ‘self-inflicted’ and various diseases (co-morbidities) affecting recipient health. Early during the pilot some respondents expressed disquiet about the employment attribute, arguing it represented unwarranted discrimination against the retired or those unemployed because of illness. We therefore asked all respondents whether this should be included. Most respondents said ‘no’, so this was omitted. We used the computer package SPEED (software designed to establish choice sets for DCEs [20]) for the pilot DCE to generate such an orthogonal main effects design. We paired choices generated by SPEED to minimize attribute overlap and level imbalance [21]. The pilot exercise analysed 60 responses to establish appropriate attributes and levels for use in the final DCE questionnaire.

#### Identifying attributes and levels for final DCE

The pilot exercise analyzed 60 responses to ascertain attributes and levels for the final DCE using Random Effects Probit. All the attributes (with the exception of the employment status attribute) proved significant at the 5% level (i.e. implying that the attribute would affect a respondent’s choice of who to prioritize for kidney transplant). Final pilot findings from attribute rankings indicated that the employment status attribute was far from highly ranked and it was therefore removed. The following inclusions were warranted, with some limited changes. Respondents thought people with dependents ought to be prioritized, but most considered adult as well as child dependents should be included, so this attribute was amended to include adults. Age was considered relevant, but the recipient age ceiling was reduced to 65 because clinicians indicated that transplantation was less likely amongst over 65s. Although highly ranked separately, when combined the life expectancy and other recipient diseases (non-CKD co-morbidities) attributes resulted in unrealistic DCE scenarios. For example, one pairwise choice resulted in respondents choosing between a 70 year old with severe arthritis with 12 years life expectancy, and a 45 year old without co-morbidities with a shorter life expectancy. Such a comparison did not make sense since a 45 year old without co-morbidities would be expected to have a longer life expectancy. So, the life expectancy attribute was replaced with one indicating whether a potential recipient had diseases predominantly affecting life expectancy. This provided more realistic scenarios and improved DCE design.

Other attributes highly ranked in the pilot exercise included ‘patient compliance’ and whether illness was ‘self-inflicted’. However, advice from medical professionals highlighted the fact that unlike ‘liver transplantation’ renal transplantation was rarely required because of alcohol or drug misuse, so this was not a particularly relevant consideration to underpin attributes and levels for the DCE. Also patients who are thought likely to abuse their bodies or be non-compliant would not be transplanted. On this basis, it was decided to exclude this attribute.

Table 1 lists the final attributes and levels selected for use in the DCE questionnaires. Copies of the questionnaires are provided in Additional file 2.

**Table 1:** **Details of final attributes and levels used for Discrete Choice Experiment** (**DCE**) **questions**

**Attribute**	**Variable name**	**Levels**	**Interpretation of coefficients**
**Time spent awaiting transplantation**	Wait	1 month, 2 years, and 10 years.	Indirect utility of each 1 year reduction in transplant recipient waiting time.
**Tissue type matching**.	Tiss	Non-favourable match: 86% average kidney survival rate post-transplant.	Indirect utility of prioritizing people for each 1% improvement in kidney survival.
		Favourable match: 89% average kidney survival rate post-transplant.	
		Perfect match: 90% average kidney survival rate post-transplant.	
**How many child or adult dependents recipients have**	Dep	None, 1, or 4 dependents.	Indirect utility of each additional dependent.
**Recipient age**	Age	20 years, 45 years, and 65 years	Indirect utility for each 1 year reduction in recipient age.
**Diseases predominantly affecting life expectancy**	dis1	No disease affecting life expectancy (other than Kidney disease) vs. moderate disease (uncontrolled hypertension or obesity) & Kidney disease.	Indirect utility of having no rather than moderate disease predominantly affecting life expectancy.
	dis2	Moderate disease (uncontrolled hypertension or obesity) affecting life expectancy vs. severe disease (heart attack, stroke, or diabetes with complications).	Indirect utility of having moderate disease rather than severe disease predominantly affecting life expectancy.
**Diseases predominantly affecting quality of life**	ill1	No disease affecting quality of life (other than Kidney disease) vs. moderate disease (mild asthma).	Indirect utility of having no disease rather than a moderate disease predominantly affecting quality of life.
	ill2	Moderate disease (mild asthma) affecting quality of life vs. severe disease (severe arthritis).	Indirect utility of having a moderate disease rather than a severe disease predominantly affecting quality of life.

The pilot questionnaire contained an explanatory preamble which described the attributes and levels included. Transplant survival rates were presented from UK Transplant and it was explained that these were contingent upon donor/recipient tissue match. Although transplant survival rates were available for longer time horizons, we wanted to avoid information overload and therefore only presented 12 month survival rates. The pilot confirmed that explanatory information was easy to understand.

#### Final Discrete Choice Experiment (DCE)

We used a binary dependent variable model in order to force a choice. This is because, in reality, transplant decisions have to be made and medical professionals face a forced choice when allocating kidneys because of donor scarcity. Moreover, pilot interviews revealed that many respondents felt uncomfortable with deciding who to transplant. Therefore, it was judged that including a ‘cannot decide’ option might have triggered such a response from people who in reality were not indifferent. An alternative would have been to allow choices between more than 2 potential recipients using a multinomial model, or to have more attributes and levels; but this would have complicated decision making [22]. Moreover, since many renal patients suffer from fatigue we wanted to avoid complicated decisions, because when complexity increases there is evidence that respondents may be more inclined to use simplifying heuristics [23] compromising response reliability. Copies of the final DCE questionnaires are provided in Additional file 2.

The final DCE design was again an Orthogonal Main Effects Plan (OMEP) design involving independent valuation of attributes. To ensure a perfectly orthogonal design and improve efficiency we used an OMEP design supplied by leading DCE designers [24] rather than SPEED as used in the pilot exercise. We also blocked 18 choices into 2 blocks of 9 questions (versions A and B) to reduce respondent fatigue and limit the patient questionnaire to 10 pages. Respondents were asked to choose between transplanting patient A with particular attribute levels or patient B with different levels (see Additional file 2 for examples).

#### Questionnaire distribution

We included an information leaflet and freepost envelope in the National Kidney Federation’s newsletter ‘Kidney Life’ (circulation c.20,000). The leaflet invited patients, carers, donors or healthcare professionals to request a DCE questionnaire. Individuals who replied were sent a questionnaire, along with a covering letter, consent form, and freepost reply envelope for the completed questionnaire. Individuals who did not return a questionnaire were not contacted again. Questionnaires were also posted directly to members of the British Organ Donor Society, healthcare professionals listed in the UK Transplant service directory, and to non-transplanting units with transplant coordinators or transplant physicians. Relatives of deceased donors were targeted via the British Organ Donation Society (BODY). To increase ethnic minority responses we provided questionnaires in a person’s preferred language. A reputable translation organization was used to translate questionnaires in Punjabi, Hindi, Bengali, Gujarati and Urdu. A bilingual researcher administered the questionnaires upon non-English speaking patients and checked the questionnaire’s translation accuracy. We obtained additional responses from Ealing NHS Trust, and from University Hospital, Coventry using translated questionnaires to increase levels of ethnic minority participation.

#### Assessing the representativeness of respondent sample

In order to assess representativeness, we used UK Renal Registry data (when available); figures were extracted for transplant success rates. Other relevant patient data were not available i.e. patients with failed transplant, awaiting a transplant, on dialysis, or with kidney disease not requiring dialysis. UK Renal Registry data also does not record detailed ethnicity information for some sub-groups of respondents, so detailed patient data is not available for all sub-groups.

#### Econometric / statistical analysis models

Two models were used for analysis (for full details refer to Additional file 3). Random Effects probit (model 1 and 2) was used to establish different stakeholder preferences. Model 1 used a series of dummy variables to ascertain whether preferences for specific attributes differed amongst carers, donors/relatives of deceased donors, and healthcare professionals compared with the patient respondents group.

Model 2 compared the preferences of ethnic minority and white majority patients. Again a series of dummy variables were used to establish whether preferences differed between ethnic minority and other patients for specific attributes.

#### Marginal Rate of Substitution (MRS)

Data were analysed in terms of a measure of marginal rate of substitution (MRS) which related differences in other attributes (potential transplant priority criteria) to waiting time for renal transplants. Analysis examined whether differences in MRS were statistically significant between stakeholder groups for specific attributes (using Wald tests). We also used the Delta method [25] with command ‘nlcom’ in STATA to estimate 95% confidence intervals. Full details of the approach used to derive MRS are provided in Additional file 4 (Table 1). Wald tests using ‘testnl’ in STATA were used to establish whether MRS differed significantly between groups; comparing patients with carers, donors and healthcare professionals (model 1); and ethnic minority patients vs. white majority patients (model 2). For example, to establish whether preferences for tissue matching differed between ethnic and non-ethnic minorities (model 2), the null hypothesis was that tissue match MRS was identical for both (p ≤ 0.05 indicated a difference at the 5% level).

## Results

### Sample characteristics

Table 2 presents respondent characteristics. We obtained 908 patient responses; 18 additional responses from Ealing NHS Trust and 5 from University Hospital, Coventry were obtained using translated questionnaires. UK Renal Registry data [26,27] was used to assess patient sample representativeness. 508/908 patient respondents (55.9%) were male, 397/908 (43.7%) were female, and 3/908 (0.3%) not reported. Renal Registry data [26] similarly indicates a slightly higher proportion of male than female patients across age groups. The average patient age in the sample was 54.9 years (median 57 years), coinciding with the Renal Registry data median age (57.3 years) [27]. Of the 895/ 908 patients indicating ethnicity, 799/895 (89.3%) were white British and 27/895 (3%) were other white background (e.g. Irish), giving a total of 92.3% white patients. UK data [26] indicates that 79.7% of renal patients are white. So white respondents are over-represented in our survey. Overall, 69/895 (7.7%) patients indicating ethnicity were non-white, compared with an expected 20.3% incidence rate [26], and 50/69 of the non-white patients were South Asians (5.6% of those indicating ethnicity) compared to an anticipated 10.5% [26].

**Table 2:** Details of characteristics of questionnaire respondent samples

	**Patients (n** = **908)**	**Carers (n**=**41)**	**Donors (n** =**48)**	**Healthcare workers (n**=**113)**
**AGE**				
**Mean age**	54.88 years	52.37 years	54.67 years	43.23 years
**GENDER**				
**Male**	508 (55.9%)	10 (24.4%)	14 (29.2%)	51 (45.1%)
**Female**	397 (43.7%)	31 (75.6%)	34 (70.8%)	61 (54.0%)
**Not indicated**	3 (0.3%)	0 (0%)	0 (0%)	1 (0.9%)
**ETHNICITY**				
**White** (**British**)	799 (88%)	38 (92.7%)	44 (91.7%)	89 (78.8%)
**White ethnic minorities**	27 (2.9%)	1 (2.4%)	1 (2.1%)	9 (8%)
**Non**-**white ethnicity** (**excluding Asians**)	19 (2.1%)	1 (2.4%)	0 (0%)	2 (1.8%)
**Non**-**white ethnicity** (**Asians**)	50 (5.5%)	1 (2.4%)	1 (2.1%)	9 (8%)
**Not indicated**	13 (1.4%)	0 (0%)	2 (4.2%)	4 (3.5%)
**DEPENDENT CHILDREN**				
**0**	755 (83.1%)	33 (80.5%)	36 (75%)	51 (45.1%)
**1**	72 (7.9%)	2 (4.9%)	5 (10.4%)	22 (19.5%)
**2**	49 (5.4%)	5 (12.2%)	2 (4.2%)	26 (23.0%)
**3**	12 (1.3%)	0 (0%)	3 (6.3%)	9 (8.0%)
>**3**	7 (0.8%)	1 (2.4%)	1 (2.1%)	3 (2.7%)
**Not indicated**	13 (1.4%)	0 (0%)	1(2.1%)	2 (1.8%)
**DEPENDENT ADULTS**				
**0**	750 (82.6%)	16 (39.0%)	39 (81.2%)	98 (86.7%)
**1**	121 (13.3%)	17 (41.5%	6 (12.5%)	11 (9.7%)
**2**	17 (1.9%)	6 (14.6%)	2 (4.2%)	3 (2.7%)
>**2**	8 (0.9%)	1 (2.4%)	0 (0%)	0 (0%)
**Not indicated**	12 (1.3%)	1 (2.4%)	1 (2.1%)	1 (0.9%)

In terms of transplant history, the patient sample comprised: 468/908 (51.5%) with successful transplants, 118/908 (13%) whose transplant had failed and 279/908 (30.7%) awaiting transplant (average wait 22.6 months). Some patients whose transplant had failed also reported they were ‘awaiting transplantation’. Of the remainder, 237/908 (26.3%) were undergoing dialysis without transplantation and 57/908 (6.3%) had kidney disease not requiring dialysis. Renal Registry data [27] indicates 46.9% of patients have successful transplants (close to our figure). There are no data for the other patient characteristics. Amongst non-whites (including Asians) our sample included 18/69 patients (26%) with successful transplants and 10/69 (14.5%) whose transplant had failed; 35/69 patients (50.7%) were awaiting a trans-plant on dialysis (average wait: 21.45 months) and 3/69 (4.3%) had kidney disease not requiring dialysis. These statistics cannot be compared with UK Renal Registry data since ethnicity is not recorded for such sub-groups [27]. However, a lower percentage figure for transplant success and a higher figure for patients awaiting transplants might be expected (ethnic minorities donate fewer organs and transplants are less likely to be closely matched).

Of the 48 donor respondents, 21 were live donors and 27 relatives of deceased donors. Healthcare professionals comprised: 9 renal surgeons, 37 renal physicians, 17 transplant co-ordinators, 31 nurses, 9 clinical scientists, 1 GP, 1 dietician, 1 network manager, 1 transplant scientist, 1 medical student, 1 transplant immunologist, 1 tissue typer, 1 clinical audit manager, 1 renal technologist and a pathologist.

### Data analysis

Table 3 presents model 1 results including MRS 95% confidence intervals (CIs). Variables are as listed in Table 1.

**Table 3:** **Model 1**: **results and MRS** (**i**.**e**. **utility value of other attributes expressed in terms of trade**-**off with 1 year waiting time**) **for patients**, **carers**, **donors**, **and healthcare workers**

**Attribute**	**Coefficient : patients**	**Implied MRS for patients**		**Coefficient : dummies for the carer group**	**Implied MRS for carers**	**Wald test p**-**values: Carers vs. patients**
**Wait**	.0443**	1		-.0156	1	
**Tiss**	.0624**	1.41*		am	0.76	p=0.0024
		(1.08 / 1.74)			(−1.42 / 2.93)	
**Dep**	.0635**	1.43*		-.0585*	0.17	p<0.0001
		(1.17 / 1.69)			(−1.54 / 1.89)	
**Age**	.0069**	0.16*		.0006	0.26	p=0.0750
		(0.12 / 0.19)			(−0.03 / 0.56 )	
**dis1**	-.0004	−0.01		.1205	4.18	p=0.2965
		(−1.03 / 1.01)			(−3.25 / 11.62)	
**dis2**	.6789**	15.32*		-.1971	16.79*	p<0.0001
		(13.45 / 17.20)			(2.69 / 30.89)	
**ill1**	-.1207**	−2.73*		.1130	−0.27	p=0.1236
		(−1.45 / -4.00)			(−9.55 / 9.01)	
**ill2**	.1850**	4.18*		-.0334	5.28	p=0.0910
		(3.12 / 5.23)			(−2.27 / 12.83)	
**Intercepts**	.1208**			-.0034		
**Attribute**	**Coefficient : dummies for the donor group**	**Implied MRS for donors**	**Wald test p-values: Donors vs. patients**	**Coefficient : dummies for the healthcare worker group**	**Implied MRS for healthcare workers**	**Wald test p-values: healthcare workers vs. patients**
**Wait**	-.0086	1		-.0039	1	
**Tiss**	-.0667*	−0.12	p<0.0001	-.0110	1.27*	p=0.0027
		(−1.62 / 1.38)			(0.24 / 2.31)	
**Dep**	-.0468*	0.47	p<0.0001	-.0003	1.56*	p=0.0017
		(−0.79 / 1.73)			(0.72 / 2.41)	
**Age**	-.0023	0.13	p=0.0067	.0127**	0.48*	p=0.0300
		(−0.05 / 0.31)			(0.31 / 0.66)	
**dis1**	.1508	4.22	p=0.1669	.1823**	4.50*	p=0.0265
		(−1.38 / 9.81)			(1.09 / 7.91 )	
**dis2**	-.2676*	11.54*	p<0.0001	.1056	19.42*	p<0.0001
		(2.90 / 20.17)			(12.71 / 26.14)	
**ill1**	.0520	−1.93	p=0.2301	.0501	−1.75	p=0.1048
		(−8.66 / 4.80)			(−5.74 / 2.24)	
**ill2**	.0245	5.87*	p=0.1942	.1790*	9.01*	p=0.9818
		(0.18 / 11.57)			(5.44 / 12.59 )	
**Intercepts**	-.112			.0844		

*: Significant at 5% level; Figures in parentheses indicate 95% confidence intervals for point estimates.

Coefficients are as indicated in Table 1. MRS in Table 3 indicates indirect utility values for changes in attributes (for direction of change see Table 1) relative to values for prioritizing a recipient waiting an extra year for transplantation (Additional file 4: Table 1 presents more detailed MRS formulae). In effect MRS indicates the rate at which the respondent group in question is willing to trade-off gains in relation to one criterion against losses in relation to another (in this case the amount of time waiting). MRS in Table 4 is expressed in terms of 1 or 5 year waiting times. Measures of ‘goodness of fit’ for model 1 (Random Effects probit) show that 63% of actual values are predicted by the model, and McFadden's R^2^ = 0.1088. A likelihood ratio test for the joint significance of the dummy variables has 27 degrees of freedom with a critical value of 40.11, compared with λ=71.90, so the dummy variables are jointly significant. The tissue match coefficient (tiss) in Table 4 indicates the impact of a 1% difference in 12 month kidney survival. Difference in survival rate between a perfect vs. favourable match is 1%, so the MRS figure of 1.41 (Table 3) also appears in Table 4 for ‘Prioritizing perfect not non-favourable tissue matches’. Table 4 indicates MRS for the ’Prioritizing someone with a favourable not non-favourable match’ (1.41 × 3 [a 3% difference in kidney survival rate] = 4.23). It also shows how much respondents value other changes in attributes compared to a 1 year wait (column 2). Moreover 5 year MRS figures are presented in Table 4 (column 3). If waiting time increases 5 fold, MRS for a 5 year wait is 1/5^th^ of 1 year MRS. 95% CIs for 5 year MRS are 1/5^th^ of 1 year. The CIs define the range within which MRS figures must lie (to be 95% confident).

**Table 4:** **Model 1**: **MRS** (**i**.**e**. **utility value of other attributes expressed in terms of trade**-**off with 1 year or 5 year waiting time**) **for patients**, **carers**, **donors and healthcare workers**

**Variable**	**Patient trade**-**off between variable &1 year wait**	**Patient trade**-**off between variable &5 years wait**	**Carers trade**-**off between variable &1 year wait**	**Carers trade**-**off between variable &5 years wait**
**Prioritizing perfect not favourable tissue matches**	1.41*	0.28*	0.76	0.15
	(1.08 / 1.74)	(0.22 / 0.35)	(−1.42 / 2.93)	(−0.28 / 0.59)
**Prioritizing favourable not non– favourable tissue matches**	4.23*	0.85*	2.27	0.45
	(3.23 / 5.22)	(0.65 / 1.05)	(−4.25 / 8.80)	(−0.85 / 1.76)
**Prioritizing a recipient with dependents – per extra dependent**	1.43*	0.29*	0.17	0.03
	(1.17 / 1.69)	(0.23 / 0.34)	(−1.54 / 1.89)	(−0.31 / 0.38)
**prioritizing a younger recipient – per year younger**	0.16*	0.03**	0.26	0.05
	(0.12 / 0.19)	(0.02 / 0.04)	(−0.03 / 0.56 )	(−.01 / 0.11)
**Prioritizing those with ‘no’ not** ‘**moderate**’ **diseases affecting life expectancy**	−0.01	0.00	4.18	0.84
	(−1.03 / 1.01)	(−0.21 / 0.20)	(−3.25 / 11.62)	(−0.65 / 2.32)
**Prioritizing those with moderate not severe diseases affecting life expectancy**	15.32*	3.06*	v16.79*	3.36*
	(13.45 / 17.20)	(2.69 / 3.44)	(2.69 / 30.89)	0.54 / 6.18)
**Prioritizing those with no not moderate diseases affecting QoL**	−2.73*	−0.55*	−0.27	−0.05
	(−1.45 / -4.00)	(−0.29 / -0.80)	(−9.55 / 9.01)	(−1.91 / 1.80)
**Prioritizing those with moderate not severe diseases affecting QoL**	4.18*	0.84*	5.28	1.06
	(3.12 / 5.23)	(0.62 / 1.05)	(−2.27 / 12.83)	(−0.45 / 2.57)
**Variable**	**Donors trade-off between variable &1 year wait**	**Donors trade-off between variable &5 year wait**	**Healthcare workers trade-off between variable &1 year wait**	**Healthcare workers trade-off between variable &5 year wait**
**Prioritizing perfect not favourable tissue matches**	−0.12	−0.02	1.27*	0.25
	(−1.62 / 1.38)	(0.32 / 0.28)	(0.24 / 2.31)	(0.05 / 0.46)
**Prioritizing favourable not non**- **favourable tissue matches**.	−0.36	−0.07	3.82*	0.76*
	(−4.86 / 4.14)	(−0.97/ 0.83)	(0.72 / 6.93)	(0.14 / 1.39)
**Prioritizing a recipient with dependents – per extra dependent**	0.47	0.09	1.56*	0.31*
	(−0.79 / 1.73)	(−0.16 / 0.35)	(0.72 / 2.41)	(0.14 / 0.48)
**prioritizing a younger recipient – per year younger**	0.13	0.03	0.48*	0.10*
	(−0.05 / 0.31)	(−0.01 / 0.06)	(0.31 / 0.66)	(0.06 / 0.13)
**Prioritizing those with no not moderate diseases affecting life expectancy**	4.22	0.84	4.50*	0.90*
	(−1.38 / 9.81)	(−0.28 / 1.96)	(1.09 / 7.91 )	(0.22 / 1.58)
**Prioritizing those with moderate not severe diseases affecting life expectancy**	11.54*	2.31*	19.42*	3.88*
	(2.90 / 20.17)	(0.58 / 4.03)	(12.71 / 26.14)	(2.54 / 5.23)
**Prioritizing those with no not moderate diseases affecting QoL**	−1.93	−0.39	−1.75	−0.35
	(−8.66 / 4.80)	(−1.73 / 0.96)	(−5.74 / 2.24)	(−1.15 / 0.45)
**Prioritizing those with moderate not severe diseases affecting QoL**	5.87*	1.17*	9.01*	1.80*
	(0.18 / 11.57)	(0.04 / 2.31)	(5.44 / 12.59 )	(1.09 / 2.52)

*: Significant at 5% level; Figures in parentheses indicate 95% confidence intervals for point estimates.

When interpreting results it might be expected that, in general, transplant preferences would lie in certain directions. On efficiency grounds improvements in kidney survival should be positively valued, and therefore respondents should generally prefer transplants with the highest chance of success. But, some stakeholder groups might not exhibit this preference if there is a lack of organs closely matching their own requirements. We might expect respondents to prioritize those waiting longer for a transplant on equity grounds, and therefore would anticipate a positive coefficient on a one year reduction in waiting time. It might also be considered that recipients with more dependents should be prioritized because more people would benefit from a recipient’s improved health. In contrast, all other things being equal, one might expect older patients to benefit less because they have a lower life expectancy, so the coefficient on reductions in recipient age would be expected to be positive. Finally, for efficiency reasons respondents might prioritize more highly those with fewer or no disease(s) affecting life expectancy over those with moderate diseases.

Patients’ MRS figures (Table 4) suggest, all other things being equal (*ceteris paribus*), that patients would prioritize recipients with perfect over favourable tissue matches (tiss) more than those waiting an extra year (1 year MRS =1.41, exceeding indirect utility from avoiding a 1 year wait of 1.00). However, if a favourably matched patient were to wait 5 years longer, they would be a higher priority than the perfect match (MRS = 0.28) <1. Similarly, prioritizing someone with a favourable not non-favourable match (*ceteris paribus*) is valued more than prioritizing someone waiting for 1 year (MRS = 4.23) as it exceeds 1, the utility from a 1 year wait. But if a potential recipient waited 5 years longer, prioritizing the longest waiter is optimal (MRS = 0.85) < 1.

Patients also prioritized someone with an extra dependent more than waiting a year longer (MRS for ‘dep’=1.43). However, if a potential recipient waited 5 years longer MRS = 0.29, for an extra dependent, so prioritizing an extra dependent is a lower priority. Prioritizing a recipient who is 1 year younger is valued less than a 1 year or 5 year reduction in waiting time (‘age’ 1 year MRS = 0.16, 5 year = 0.03). Patients would also not prioritize those with no vs. moderate diseases predominantly affecting life expectancy (‘dis 1’ is insignificant), but would prioritize (dis2) those with moderate rather than severe diseases predominantly affecting life expectancy highly (1 year MRS = 15.32; 5 year MRS = 3.06). Thus, someone with a moderate, not severe, disease predominantly affecting life expectancy is prioritized (MRS >1).

Paradoxically, for diseases predominantly affecting quality of life, rather than life expectancy, patients prioritized those with moderate not no disease (‘ill1’ has a 1 year waiting time MRS of −2.73), perhaps because many patients have moderate co-morbid diseases. However, 5 year MRS equals −0.55, so long waiters are a higher priority than those with moderate rather than no disease affecting quality of life. Finally, patients prioritized those with moderate rather than severe diseases predominantly affecting quality of life (‘ill2’ 1 year MRS = 4.18; 5 year MRS = 0.84). So, ceteris paribus, someone with moderate not severe disease would be a higher priority than someone waiting 1 year longer (MRS>1), but a lower priority than someone waiting 5 years longer (MRS < 1).

Carer results (Tables 3 and 4) were compromised to some extent by the smaller sample size (n=41), so MRS was only significant for 1 variable - prioritizing those with dependents (dep) (Table 3, column 5). The fact that other MRS figures are insignificant may partly be attributable to the size of sample. Wald test results (Table 3, column 7) indicate statistically significant differences in MRS between other stakeholder groups and patients (5% level). The results presented in Table 4 suggest MRS for prioritizing perfect over favourable tissue matches is lower amongst carers than patients (1 year MRS = insignificant vs. 1.41; 5 year MRS = insignificant vs. 0.28); and lower for prioritizing favourable over non-favourable matches (1 year MRS = insignificant vs 4.23; 5 year MRS = insignificant vs. 0.85). Moreover, Wald tests (Table 3, column 7) show carers’ preference for prioritizing those with dependents is less than patients’ (1 year MRS = insignificant vs. 1.43; 5 year MRS = insignificant vs. 0.29). Wald tests also show that carers prioritize those with moderate not severe diseases predominantly affecting life expectancy (dis2) more than do patients (1 year MRS = 16.79 vs. 15.32; 5 year MRS = 3.36 vs. 3.06).

Donor response analysis was also compromised by a smaller sample size (n=48). This may explain why MRS (MRS for 1 and 5 years in Table 4) is only significant for 2 variables (‘dis2’ and ‘ill2’). Once again, Wald tests (Table 3, column 4) suggest that donors value tissue match (tiss) less than patients (1 year MRS = insignificant vs. 1.41; 5 year MRS = insignificant vs. 0.28) for perfect not favourable matches, and also value favourable not non-favourable matches less (1 year MRS = insignificant vs. 4.23; 5 year MRS = insignificant vs. 0.85). Similar to carers, Wald tests (Table 3, column 4) indicate donors value prioritizing dependents (dep) less than patients do (1 year MRS = insignificant vs. 1.43; 5 year MRS = insignificant vs. 0.29). They also suggest that donors value prioritizing the young (age) less than patients (1 year MRS = insignificant vs. 0.16; 5 year MRS = insignificant vs. 0.03). Donors, in contrast to carers, prioritize those with moderate rather than severe co-morbidities predominantly affecting life expectancy less than do patients (1 year MRS = 11.54 vs. 15.32; 5 years MRS = 2.31 vs. 3.06). Wald tests do not indicate other differences.

Analysis of healthcare professionals’ responses indicates MRS is significant for 6/7 variables (Tables 3 and 4) and Wald tests (Table 3, column 7) suggest healthcare professionals’ preferences differ from those of patients for 5/7 variables. Professionals value prioritizing those with better tissue matches ‘tiss’ less than do patients (1 year MRS = 1.27 vs. 1.41; 5 year MRS = 0.25 vs. 0.28) for perfect not favourable matches, and prioritize favourable vs. non-favourable matches less (1 year MRS = 3.82 vs. 4.23 ; 5 year MRS = 0.76 vs. 0.85). Wald tests also indicate healthcare professionals prioritize those with dependents (dep) more (1 year MRS = 1.56 vs. 1.43; 5 year MRS = 0.31 vs. 0.29), and younger recipients (age) more (1 year MRS = 0.48 vs. 016; 5 year MRS = 0.10 vs. 0.03). They would also prioritize (dis1) those with no vs. moderate diseases affecting life expectancy whereas patients would not (1 year MRS = 4.50 vs insignificant; 5 year MRS = 0.90 vs. insignificant). Similarly, healthcare professionals also prioritized (dis2) those with moderate rather than severe diseases affecting life expectancy more than patients did (1 year MRS = 19.42 vs. 15.32; 5 year MRS = 3.88 vs 3.06). However, there was no evidence that healthcare professionals would prioritize recipients with co-morbid diseases predominantly affecting quality of life differently from patients (Wald tests for ill1 and ill2 are insignificant). Importantly, the fact that healthcare professionals exhibit statistically significant differences to patients for 5/7 variables suggests that, if healthcare professionals’ preferences were to prevail in transplant decision-making, this could result in transplant allocation decisions which inadequately reflect patient preferences.

Measures of ‘goodness of fit’ for model 2 indicate 62.09% actual values are predicted by the model, and McFadden's R^2^ = 0.133. A likelihood ratio test for the significance of the dummy variables has λ = 35.83, which compares with a critical value for 9 degrees of freedom of 16.92, so the dummy variables are jointly significant.

Table 5 compares ethnic minority and white majority patients (model 2). Coefficients are as defined in Table 1, and MRS specified in Additional file 4. Overall 3 dummy variables (tiss, dep and dis2) were significant, but Wald tests (Table 5, column 6) suggest more differences including the following. Ethnic minorities do not prioritize recipients with better tissue matches (tiss) but the majority population do (1 year MRS = insignificant vs. 1.54; 5 years MRS = insignificant vs. 0.31 for perfect rather than non-favourable matches). For favourable, rather than non-favourable, matches only white majority patients valued favourable matches significantly (1 year MRS = insignificant vs. 4.64; 5 years MRS = insignificant vs. 0.93). This is perhaps because ethnic minorities are disadvantaged if a close tissue match is required, due to a lack of ethnic minority donors. Wald test results indicate that MRS for prioritizing younger (age) rather than older recipients differs only marginally between ethnic minority and other patients (1 year MRS = 0.15 vs. 0.16; 5 year MRS = 0.03 vs. 0.03). Finally, Wald tests also suggest ethnic minority patients value prioritizing recipients with moderate vs. severe diseases (dis2) affecting life expectancy less than other patients (1 year MRS = 10.25 vs. 15.86; 5 year MRS = 2.05 vs 3.17). Once again, this is perhaps linked to the higher prevalence of severe diseases / co-morbidities predominantly affecting life expectancy amongst ethnic minorities. Wald tests did not indicate that valuation of other attributes varied by ethnicity.

**Table 5:** **Model 2**: **Patient values vs**. **those of ethnic minorities** (**96 out of 908 are ethnic minorities)**

**Variable**	**Coefficient for non-ethnic minorities**	**MRS for non-ethnic minorities**	**Coefficient for dummy variables for ethnic minorities**	**MRS for ethnic minority patients**	**Wald test p**-**values**
**Wait**	.0451*	1	-.0061	1	
**Tiss**	.0698*	1.54*	-.0630**	0.17	p<0.0001
		(1.19 / 1.90)		(−0.82 / 1.17)	
**Dep**	.0595*	1.32*	.0351*	2.42*	p=0.2755
		(1.05 / 1.59)		(1.40 / 3.44)	
**Age**	.0071*	0.16*	-.0011	0.15*	p=0.0024
		(0.12 / 0.20)		(0.03 / 0.27)	
**dis1**	.0039	0.09	-.0398	−0.92	p=0.6014
		(−0.98 / 1.15)		(−4.41 / 2.57)	
**dis2**	.7158*	15.86*	-.3153**	10.25*	p<0.0001
		(13.87 / 17.85)		(4.96 / 15.53)	
**ill1**	-.1085*	−2.40*	-.0903	−5.08*	p=0.9050
		(−1.06 / -3.74)		(−0.83 / -9.33)	
**ill2**	.1773*	3.93*	.0647	6.19*	p=0.2558
		(2.82 / 5.03)		(2.51 / 9.88)	
**Intercepts**	.1269*		-.0510		
**Variable**	**Non**-**ethnic minorities trade**-**off between variable &1 year wait**	**Non**-**ethnic minorities trade**-**off between variable &5 year wait**	**Ethnic minority trade**-**off between variable &1 year wait**	**Ethnic minority trade**-**off between variable &5 year wait**	
**Prioritizing perfect not favourable tissue matches**	1.54*	0.31*	0.17	0.35	
	(1.19 / 1.90)	(0.24 / 0.38)	(−0.82 / 1.17)	(−0.16/ 0.23)	
**Prioritizing favourable not non**- **favourable tissue matches**.	4.64*	0.93*	0.52	0.10	
	(3.57 / 5.70)	(0.71 / 1.14)	(−2.46 / 3.50)	(−0.49 / 0.70)	
**Prioritizing a recipient with dependents – per extra dependent**	1.32*	0.26*	2.42**	0.48*	
	(1.05 / 1.59)	(0.21 / 0.32)	(1.40 / 3.44)	(0.28 / 0.69)	
**prioritizing a younger recipient – per year younger**	0.16*	0.03*	0.15*	0.03*	
	(0.12 / 0.20)	(0.02 / 0.04)	(0.03 / 0.27)	(0.01 / 0.05)	
**Prioritizing those with no not moderate diseases affecting life expectancy**	0.09	0.02	−0.92	−0.18	
	(−0.98 / 1.15)	(−0.20 / 0.23)	(−4.41 / 2.57)	(−0.88 / 0.51)	
**Prioritizing those with moderate not severe diseases affecting life expectancy**	15.86*	3.17*	10.25*	2.05*	
	(13.87 / 17.85)	(2.77 / 3.57)	(4.96 / 15.53)	(0.99 / 3.11)	
**Prioritizing those with no not moderate diseases affecting QoL**	−2.40*	−0.48*	−5.08*	−1.02*	
	(−1.06 / -3.74)	(−0.21 / -0.75)	(−0.83 / -9.33)	(−0.17 / -1.87)	
**Prioritizing those with moderate not severe diseases affecting QoL**	3.93*	0.79*	6.19*	1.24*	
	(2.82 / 5.03)	(0.56 / 1.01)	(2.51 / 9.88)	(0.50 / 1.98)	

*: Significant at the 5% level; Figures in parentheses indicate 95% confidence intervals for point estimates.

Includes MRS expressed in terms of utility value of other attributes expressed in terms of trade-off with 1 year and 5 year waiting time.

## Discussion

This study is unique because, although DCEs have been used in relation to liver transplantation to identify public [17] and patient [16] preferences, this is the first application of DCEs exclusively relating to prioritizing renal transplants. Moreover, the detailed comparisons between stakeholder respondent groups are unprecedented.

Usually when DCEs are used to address healthcare issues they look at patient preferences. In contrast our study compares preferences across a range of different stakeholder groups, deploying a new approach that had not been deployed in kidney transplant research before this project. The DCE approach allows for comparison of preferences between groups, and assessment of whether differences are statistically significant. Importantly, our findings indicate when stakeholder groups’ preferences differ. This means that DCE studies that only elicit preferences for one group may fail to take into account preference heterogeneity. Establishing whether preferences vary between stakeholder groups (especially patients and healthcare professionals) is important for policy and practice.

Although DCEs are increasingly used in health services research, one potential limitation can be the sensitivity of results to the choice of attributes presented, since it is only possible to indicate trade-offs in relation to the actual attributes selected. Therefore, it is essential to consult a wide range of opinion during the attribute selection process, including patients and professionals, before deciding upon which attributes to include. The present study included such a process. Constructing a robust DCE also requires that the choice of attributes has emerged from a thorough pilot exercise. In the present study, a great deal of time was invested in piloting the questionnaire to try to ensure that the range of attributes and levels identified for inclusion in our DCE questionnaires was appropriate.

Our analysis of *patient* responses showed that respondents valued prioritizing patients with closer tissue matches, but also valued other factors significantly including prioritizing: long waiters; those with child or adult dependents; and younger recipients. Furthermore, in terms of co-morbidities affecting life expectancy, individuals with moderate diseases were prioritized over those with severe diseases, but those with moderate diseases were not prioritized over those with no such disease. In terms of diseases predominantly affecting quality of life (rather than life expectancy) patients prioritized recipients with moderate rather than no disease, and those with moderate rather than severe disease. However, for *ethnic minority patients* our findings demonstrate that, unlike other patients, this group did not value tissue match significantly. They also valued prioritizing those with severe rather than moderate disease affecting life expectancy less than other patients did.

We are also able to report on the preferences of *carers*. Although the sample was small (n = 41), it was sufficient to establish some statistically significant differences when compared with patients’ responses, but probably insufficient for all differences in preferences between carers and patients to be demonstrated in a statistically significant manner. The number of carer responses obtained via our request in the publication ‘Kidney Life’ was probably limited by the fact that this publication is read more by patients than those who care for renal patients. An alternative strategy would have been to ask patient respondents to supply the name and address of their carer (if applicable) to approach. However, despite the fact we only had 41 carer responses, this data was sufficient to establish that some *carer* preferences differ significantly from those of patients. In contrast to patients, carers did not value prioritizing those with better tissue matches or those with dependents. But, they did value prioritizing those with moderate not severe diseases affecting life expectancy more than patients. Whilst it is interesting that carer preferences differed from those of patients, patient preferences are clearly more important in terms of decisions on kidney transplant criteria.

In terms of *donor* preferences, the sample size (n = 48 *donor families* / *live donors*) was sufficient to discern that some preferences differed in a statistically significant manner compared to those of patients. Our findings indicate that donors, like ethnic minority patients, did not value prioritizing better tissue matches significantly. They also valued transplants to those with dependents, younger recipients, and those with moderate rather than severe disease affecting life expectancy more than patients did. Donor preferences are important to establish because without donors transplantation programmes cannot continue. It might have been possible to obtain a larger sample of this stakeholder group if we had targeted people on the organ donor register as well as actual donor families and live donors, this would have increased statistical power thereby potentially allowing us to establish other statistically significant differences in preferences.

The number of responses from *healthcare professionals* (n = 113) was more than adequate to discern preferences for the group as a whole. In terms of overall preferences, healthcare professionals’ preferences differed from those of patients in that professionals valued prioritizing better tissue matches less than patients did, but valued prioritizing those with dependents more. They also prioritized those with no rather than moderate diseases predominantly affecting life expectancy whereas patients would not; and prioritised those with severe rather than moderate diseases affecting quality of life more than patients. Unfortunately, the sub-sample of renal physicians (as opposed to healthcare professionals more generally) was not large enough to establish how their preferences might differ from those of patients. Given that it is renal physicians who are involved in decisions about allocating kidneys, more detailed information on the preferences of this important group of healthcare professionals would have been useful.

The difference between patients and healthcare professionals in prioritizing recipients with diseases affecting quality of life may be rationalized if patient preferences are biased due to many individuals in the patient group having moderate disease. However, it is less clear why healthcare professionals place less emphasis upon closeness of donor–recipient tissue match. In this respect, our findings indicate that if transplant allocation decisions and policies are based solely on healthcare professionals’ own preferences this may conflict with patient wishes.

In terms of relevance for transplant policy, our DCE study was not intended to identify specific *individuals* “who should be prioritized for renal transplantation”. Rather, it aimed to identify certain potential characteristics of kidney recipients characteristics which different stakeholder groups consider should be prioritized, and therefore suggest potential transplant recipient *groups* who ought to be made a higher or lower priority for transplantation. It is reassuring that our findings are broadly supportive of the 2006 revisions to UK kidney transplant policy in terms of prioritizing long waiters and young adults. However, although our analysis shows that this can be justified, it also suggests that other criteria (i.e. prioritizing those with dependents) ought to be considered.

Our findings can be considered alongside a number of earlier non-DCE studies. An Australian-based renal study unlike ours adopted a general public perspective [10]. Respondents were found to prioritize long waiters and the young, but had a split verdict over whether to prioritize those with children. Similarly, renal research into African Americans’ preferences [9] indicates that kidney allocation based upon HLA matching is considered unfair. However, at the same time, African Americans did not want to receive organs with lower survival rates; note since this paper was published (1997) graft survival for poorer matches has improved. More recently, a 2005 Glasgow renal study [11] has used a non-DCE scenario approach to consider allocation of deceased donor kidneys for transplantation. Interestingly, certain findings from this research conflict with our results (i.e. tissue matching was not a major allocation criterion) although, like our findings, the researchers reported that emphasis was placed on prioritizing long-waiters (albeit defined by time on dialysis, not on waiting lists). One DCE study, a 2010 Canadian article on patients with chronic kidney disease [19] has reported that respondents preferred to prioritize kidney transplants on the basis of a ‘best match’ rather than ‘first come, first served.’ However, in contrast to our study, this particular DCE considered a wide range of attributes relating to CKD in general (including organ procurement and the organization of care) and as such could provide only a very limited indication of preferences for kidney transplant allocation. The DCE included only one attribute relating to kidney transplants (“How should deceased donor kidneys for transplantation be allocated for transplantation”) with just two possible levels ‘best match’, or ‘first come, first served.’ Moreover, unlike our DCE study which furnished respondents with information on the likelihood of kidney transplants being successful for non-favourable matches, it is unclear whether similar information was provided in the Canadian study to ensure fully informed responses.

Interestingly, a recent article which discusses new allocation concepts [28] emphasises efficiency criteria related to maximising health gains (i.e. getting the most life years from organs available for transplant). Whilst maximising life years or Quality Adjusted Life Years (QALYs) may be a legitimate transplant policy objective (and is supported by our findings in the sense that respondents value prioritizing younger patients in our DCE analysis), it is clear from our results that stakeholders also value equity considerations (i.e. avoiding patients having to face excessive waiting times). This is something which comes out strongly in our analysis of different stakeholders’ priorities, but would be neglected in an approach which focuses upon maximising health gains. Furthermore, the findings reported here and in our earlier analysis [18] indicate that, although both time spent waiting and the quality of tissue match between donor and recipient are of importance to healthcare workers and patients, amongst ethnic minority patients closeness of tissue match is not a significant determinant of patient preferences.

The findings reported in this paper, and those reported in our earlier analysis [18], suggest that both time spent waiting and the quality of tissue match between donor and recipient are of importance to healthcare workers and to non-ethnic minority patients, but that amongst ethnic minority patients closeness of tissue match is not a significant determinant of patient preferences. As DCEs can be used in order to quantify key stakeholders’ willingness to ‘trade-off’ between conflicting transplant allocation criteria, data from the present study could in principle be used to underpin kidney transplant allocation policy thereby increasing transparency [29]. For example, if the weightings obtained were to be used for informing organ allocation decisions then, rather like the MELD scores which underpin Liver transplant policy in the USA, this would increase transparency. However, such an approach would sideline other valid evidence [3,9-11,28,30] resulting in a more mechanistic process. In our view therefore such a mechanistic approach is inappropriate.

The United Network for Organ Sharing (UNOS) policy takes into account the length of time spent on the waiting list; whether the potential organ candidate is a child; body size of both donor and candidate; tissue match between donor and candidate; blood type; and blood antibody levels. Although we wanted to avoid being too prescriptive about how either UK or USA transplant policy should be changed, in the light of our findings it is clear that changes to USA transplant policy in 2003, and UK transplant policy in 2006, have already led to a shift away from considerable reliance upon transplanting on the basis of a close HLA tissue match between donor and recipient. Changes to USA policy (7^th^ May 2003) involved an elimination of HLA-B similarity as a transplant allocation criterion [30]. This was because improvements to medications used to prevent transplant rejection reduced the benefit that previously had been associated with HLA-B matching (which had discriminated [perhaps unintentionally] against ethnic minorities). The current US policy has been suitably characterized as one of “Equal opportunity supplemented by fair innings” [31] and reported to have “improved access to transplantation for all minority groups” in the USA [28]. The first 6 year follow-up reported that the 2003 change in policy “has improved access to transplantation for all minority groups and has not been associated with a decrease in 2 year graft survival” [30]. Decreased emphasis on close tissue matching and more emphasis on prioritizing long waiters is similarly reported to have reduced the extent to which ethnic minority groups are disadvantaged in the UK [18].

However, our finding that UK ethnic minority patients do not value prioritizing recipients on the basis of closeness of tissue match indicates that there may be scope in the USA and the UK to consider further reducing the reliance upon donor-recipient HLA matching when allocating kidneys, without triggering a reduction in overall rates of graft survival if rates of graft survival continue to improve anyway. Such a policy shift would mean that the preferences of ethnic minority patients are better accommodated by transplant policy, alongside the preferences of other patients. More recently in 2011 [32] a USA concept document has been launched relating to kidney transplantation which advocates prioritizing the young because they have greater capacity to benefit. A problem with the approach is that whilst it might help maximize overall life expectancy from available transplants it discriminates against the old [32]. However, children are normally treated as an absolute priority, and our DCE analysis shows nothing to suggest otherwise. Indeed our findings indicate that this preference extends to young adults, which is not a completely new finding but important to make clear.

A further step in extending the differentiation between patients on the transplant list would be to include social and medical factors as well as age. The issue of whether an allocation policy should treat people differently, either because they are felt to be more ‘deserving’ or because allocating organs to some people and not others will give longer graft survival overall, is part of the equality/efficiency debate [3,18]. In this respect our study makes an important contribution. Our pilot study ruled out a preference for social factors such as prioritizing those in employment. Our main DCE findings did show a preference for allocation according to some co-morbidities affecting life expectancy or quality of life. Our research also indicates that a measure of ‘social value’ (whether recipients have dependents) was valued by UK respondents. So, when transplant policy is re-appraised, consideration might be given to this additional criterion, though it is equally possible that the transplant policy group might not wish to include it for practical or ethical reasons. In either case, the use of DCEs to define and quantify stakeholders’ preferences can provide a valid structure for the decision making process.

## Conclusions

The findings of this study raise significant issues around transplant allocation to those from ethnic minority groups (who unlike other patients do not favour allocation on the basis of a close tissue match between donor and recipient; and also value prioritizing those with severe rather than moderate disease affecting life expectancy less than other patients do). Our findings also highlight important differences in preferences between healthcare professionals and patients (healthcare professionals prioritize better tissue matches less than patients do, but value prioritizing those with dependents more). Professionals would also prioritize those with no rather than moderate co-morbidities affecting life expectancy (more than patients), and would prioritize severe rather than moderate co-morbidities affecting quality of life more than patients would). Whilst respondents in our study did not think employment status should be a factor in kidney allocation, having dependents was valued. These findings ought to be considered when UK renal transplant policy is next re-appraised. This research also adds to the growing international literature relating to transplant allocation policy.

## Abbreviations

BODY: British Organ Donation Society;CI: Confidence Interval;CKD: Chronic Kidney Disease;HLA: Human Leucocyte Antigen;DCE: Discrete Choice Experiment;LREC: Local Research Ethics Committee;MRS: Marginal Rate of Substitution;OMEP: Orthogonal Main Effects Plan;QALY: Quality adjusted life year;UK: United Kingdom;UNOS: United Network for Organ Sharing

## Competing interests

There are no conflicts of interests between the independence of the authors’ contributions and the source of funding. The study was funded by the Coventry Kidney Fund.

## Authors’ contributions

Mr MC (Senior Research Fellow – Health Economics), wrote the paper which was amended in the light of co-authors and peer reviewers feedback. He managed the research project, and conducted the final data analysis. Mr Clark designed the pilot DCE questionnaire using the computer package SPEED under the oversight of Dr Julie Ratcliffe (a DCE expert who had undertaken published DCE work relating to Liver transplantation) and conducted most of the pilot DCE research. He then worked on the final design of the DCE with the support of Dr Julie Ratcliffe, and leading statisticians in the field of DCE design (Dr Street , and Dr Burgess – see reference [24]) who provided the final DCE design template. He then undertook the UK national DCE survey, and analyzed the data. Professor DL (Department of Economics, University of Warwick) provided guidance in relation to applying appropriate econometric methods to underpin the Econometric methods deployed in this paper. In particular, Professor Leech suggested the use of the Delta method to establish the statistical significance or otherwise of Marginal Rates of Substitution (MRS), and suggested the use of Wald tests to establish whether there are statistically significant differences in MRS between different stakeholder groups. Dr AG (Honorary Fellow, Warwick Medical School), conducted pilot interviews upon some non-English speaking ethnic minority patients. He arranged for the questionnaires to be translated into other languages for non-English speaking respondents, and then checked translations before distributing questionnaires to non-English speaking survey respondents who requested a questionnaire. In the interests of boosting final responses from ethnic minority groups he also interviewed some non-English speaking respondents at University Hospital, Coventry, and Ealing Hospital who completed a final questionnaire. He also contributed to the final paper. Dr DM (Research Fellow, Third Sector Research Fellow, University of Birmingham), introduced Mr Clark to the use of STATA, and STATA do-files in order that Mr Clark could conduct the econometric analysis contained in this paper. He then contributed to the final draft of the paper. Professor AS (Co-Director of the Centre for Evidence in Health and Diversity [CHEED]), contributed to the writing of the paper and management of the research, and advised on ethnic minority / diversity issues. NW (Transplant co-ordinator, University Hospital, Coventry), oversaw the distribution of final questionnaires to healthcare professionals, and commented upon the draft of the paper prior to publication. Dr RH (Renal Consultant, University Hospital, Coventry) commissioned this research and recognised the potential of deploying discrete choice experiment analysis to analyse data relating to different stakeholder group preferences for renal transplantation. He has also suggested a range of changes to the paper and assisted in making it clinically relevant. All authors read and approved the final manuscript.

## Pre-publication history

The pre-publication history for this paper can be accessed here:

http://www.biomedcentral.com/1471-2369/13/152/prepub

## Supplementary Material

Additional file 1 **Further details on the pilot exercise.** Description: This file provides a more comprehensive description of the pilot exercise undertaken. Click here for file

Additional file 2 **Copies of DCE questionnaires.** Description: This file contains the questionnaires used in the DCE study. Copies of the following are provided:
●Questionnaire Version 4a: Patient version.
●Questionnaire Version 5b: Carer version.
●Questionnaire Version 6a: Healthcare professional version.
●Questionnaire Version 7b: Donor / relative of deceased donor version. Click here for file

Additional file 3 **Technical details about the econometric models (Model 1 and Model 2) used to establish stakeholder preferences ‘Econometric / statistical analysis.’** Description: This file provides a more detailed description of the econometric models used to underpin point 6 of the ‘Methods’ section. Click here for file

Additional file 4 **Deriving Marginal Rate of Substitution (MRS) for attributes with respect to waiting time.** Description: This file provides information on how marginal rates of substitution (point 7 of the ‘Methods’ section) were calculated using data obtained from econometric models 1 and 2 (outlined in Additional file 3). Click here for file
