# Supplementary material for: Who should be prioritized for renal transplantation?: Analysis of key stakeholder preferences using discrete choice experiments
Source: BMC Nephrol. 2012 Nov 22;13:152. doi: 10.1186/1471-2369-13-152 (PMC3576250; doi:10.1186/1471-2369-13-152)
Supplement: Additional file 1 — Further details on the pilot exercise. Description: This file provides a more comprehensive description of the pilot exercise undertaken. [file 1471-2369-13-152-S1.doc]

# Additional files

### Additional file 1 – Further details on the pilot exercise

We interviewed 60 respondents (who completed questionnaires and ranked potential attributes) to inform attribute and level selection. Respondents included 41 patients (including 8 ethnic minorities), 16 healthcare professionals, 1 donor, 1 carer, and a renal Consultant’s secretary).

They completed a DCE questionnaire, and ranked attributes (as described in the questionnaire and written on cards) in priority order. Pilot respondents could also suggest other potential attributes, and details of these were written on cards. They then were asked to place cards in order of priority.

Most respondents (n = 56) came from the University Hospital, Coventry, including 4 ethnic minorities. Another 4 ethnic minority patients came from Ealing NHS Trust (to boost minority responses).

Pilot DCE attributes and levels included: waiting time (levels: 1 month, 2 years, or 10 years); tissue match (levels: non-favourable, favourable, and perfect,); employment status (levels: unemployed, part-time, or full time); number of dependent children or adults (levels: 0, 1, or 4); extra years of life expectancy (levels: 1, 5 or 12 years); recipient age (levels: 20, 45, or 70 years); and other recipient diseases (levels: healthy except for kidney disease, kidney disease plus a condition affecting activities [asthma], and kidney disease plus a condition affecting daily activities [severe arthritis]).
