# Supplementary material for: Who should be prioritized for renal transplantation?: Analysis of key stakeholder preferences using discrete choice experiments
Source: BMC Nephrol. 2012 Nov 22;13:152. doi: 10.1186/1471-2369-13-152 (PMC3576250; doi:10.1186/1471-2369-13-152)
Supplement: Additional file 2 — Copies of DCE questionnaires. Description: This file contains the questionnaires used in the DCE study. Copies of the following are provided: ●Questionnaire Version 4a: Patient version. ●Questionnaire Version 5b: Carer version. ●Questionnaire Version 6a: Healthcare professional version. ●Questionnaire Version 7b: Donor / relative of deceased donor version. [file 1471-2369-13-152-S2.doc]

Who should be prioritized for Kidney Transplants in the UK?

## A SURVEY OF YOUR PREFERENCES.

REF:

Undertaken by:


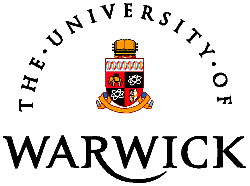


Warwick Medical School,

University of Warwick,

Coventry,

UK,

CV4 7AL.

Undertaken for:

University Hospitals of Coventry

and Warwickshire NHS Trust,

Walsgrave Hospital,

Clifford Bridge Road,

Coventry,

CV2 2DX

**Version 4a: Patient version**

# What is this questionnaire about?:

In this questionnaire we are trying to establish how to prioritize patients for a renal transplant. It is important for us to ensure that the systems for kidney allocation take account of the views of patients, health professionals and others involved in the transplant process. This questionnaire is being distributed as part of a research project aimed to provide increased information about what criteria should be used in order to allocate kidney transplants, and what weight should be attached to different criteria. This is independent research, which may in time have an impact upon kidney allocation policy. It will take into account the views of key stakeholders who may have an interest in the criteria to be used to prioritize transplants. Stakeholders will include renal patients; healthcare workers routinely working with renal patients; those caring for renal patients; kidney donors and relatives of deceased donors.

In order to do this we have developed this questionnaire. The questionnaire is trying to find out your preferences for a number of different hypothetical (illustrative) health care scenarios for treatment of patients who need a renal transplantation. Please begin by familiarising yourself with the various possible alternatives by **reading the box below.** Please assume that all other factors other than those which we indicate may differ, are equal.

Background information that we can provide you with which may inform your choices is as follows:

- This questionnaire relates only to allocation from deceased (dead) donors, currently there are over 5,000 people awaiting a transplant in the UK, and this year about 1,300 will get a deceased donor transplant.
- Under the matching system currently used in the UK, it is possible to get a kidney transplant at any time after going on the list, but someone may wait for many years and occasionally someone gets a transplant after being on the list for only a few weeks.
- The main factors used to allocate kidneys are waiting time and tissue matching. This means that people with rare or unusual tissue types have reduced chance of getting a cadaveric transplant. This applies to many people from ethnic minorities.
- A difference in tissue type between the donor and recipient was the main cause of transplant rejection in the past. However, with better anti-rejection drugs, rejection is not now the main cause of transplant loss, though a kidney transplant with an excellent tissue type now has a slightly better chance of survival than one with a reasonable match (Figures from UK Transplant: 1 year survival for perfect match it is 90%; for favourable match it is 89%; and for a non-favourable match it is 86%).

**Different criteria for prioritizing transplants**

| **Description of alternatives.**  **A) Amount of time a person has waited.**  Timescales for people receiving a transplant after being placed on a waiting list are likely to differ. The waiting time could be:  1) 1 month.  2) 2 years.  3) 10 years.  **B) Tissue type matching**  – and likelihood of transplant success.  This affects the likelihood of a transplant proving to be successful. Below are the up to date figures from UK Transplant for the survival of all transplants in the UK. There are 6 main tissue types used in matching. A perfect tissue type match is all 6 types matching; favourable is 4-5 out of 6 matching, non-favourable is less than 4 matching. If a transplant fails the patient  will return to renal dialysis.  1) Non-favourable tissue match (86% average survival rate of the kidney 1 year after the transplant).  2) Favourable tissue match (89% average survival rate of the kidney 1 year after the transplant).  3) Perfect tissue type match (90% average survival rate of the kidney 1 year after the transplant).  **C) How many dependents (either children or adults) recipients have.**  Some respondents might consider that those who have dependent children, or others who are dependent either because of their age or a physical or mental handicap, ought to be prioritized for kidney transplant. So we assume that respondents might have:  1) No dependents.  2) 1 dependent.  3) 4 dependents.  **D) Recipient age.**  The recipient could be aged either:  1) 20 years.  2) 45 years.  3) 65 years  **E) Diseases affecting life expectancy.**  As well as having kidney failure, someone may have other conditions prior to kidney transplantation which affect their life expectancy. Some of the conditions which reduce life expectancy may occur in young people, and some older people may be entirely healthy apart from kidney disease. We assume these could be either:  1) None.  2) Moderate diseases (uncontrolled hypertension or obesity).  3) Severe diseases (heart attack, or stroke, or diabetes with complications).  **F) Other recipient illnesses.**  Someone with kidney failure may have conditions other than kidney failure which are not life-threatening but do affect their quality of life. Respondents might or might not wish to allocate kidneys according to such conditions. Examples would be:-  1) Healthy except for kidney disease.  2) Kidney disease with a condition that sometimes affects their activities, such as mild asthma.  3) Kidney disease with a condition that affects their activities on a daily basis, such as severe arthritis. |
| --- |

##### INSTRUCTIONS FOR COMPLETION OF THE QUESTIONNAIRE

- We now want you to choose between different options. Please remember that the questions are hypothetical, but we want you to assume that what the questions tells you is what is actually happening.
- Everything else about the pattern of who receives what care apart from the stated differences is identical.
- Please answer every question remembering that there are no right or wrong answers, it is finding out what your personal preferences are that matters.
- Assume there is 1 kidney that could be transplanted to either patient A or patient B.

NOW PLEASE READ DESCRIPTIONS OF OPTION A AND B AND INDICATE WHO YOU THINK SHOULD BE PRIORITISED FOR A KIDNEY TRANSPLANT – PATIENT A OR PATIENT B?:

| 1 | Patient A | Patient B |
| --- | --- | --- |
| Amount of time a person has waited for a transplant | 1 month | 2 years |
| Tissue type match – and likelihood of transplant success. | 86% average 1 year chance of transplant success | 89% average 1 year chance of transplant success |
| **How many dependents (children or adults) recipients have.** | No dependents | 1 dependent |
| **Recipient age** | 20 years | 45 years |
| Diseases affecting life expectancy | None | Moderate: Uncontrolled hypertension or obesity |
| Other recipient illnesses (other than Kidney disease) | None | Mild asthma |

Which patient would you choose? Patient A **❑** Patient B **❑**

(tick 1 box only)

| 2 | Patient A | Patient B |
| --- | --- | --- |
| Amount of time a person has waited for a transplant | 2 years | 10 years |
| Tissue type match – and likelihood of transplant success. | 89% average 1 year chance of transplant success | 90% average 1 year chance of transplant success |
| **How many dependents (children or adults) recipients have.** | 1 dependents | 4 dependents |
| **Recipient age** | 45 years | 65 years |
| Diseases affecting life expectancy | Moderate: Uncontrolled hypertension or obesity | Severe: Heart attack, or stroke, or diabetes with complications. |
| Other recipient illnesses (other than Kidney disease) | Mild asthma | Severe arthritis |

Which patient would you choose? Patient A **❑** Patient B **❑**

(tick 1 box only)

| 3 | Patient A | Patient B |
| --- | --- | --- |
| Amount of time a person has waited for a transplant | 10 years | 1 month |
| Tissue type match – and likelihood of transplant success. | 90% average 1 year chance of transplant success | 86% average 1 year chance of transplant success |
| **How many dependents (children or adults) recipients have.** | 4 dependents | No dependents |
| **Recipient age** | 65 years | 20 years |
| Diseases affecting life expectancy | Severe: Heart attack, or stroke, or diabetes with complications | None |
| Other recipient illnesses (other than Kidney disease) | Severe arthritis | None |

Which patient would you choose? Patient A **❑** Patient B **❑**

(tick 1 box only)

| 4 | Patient A | Patient B |
| --- | --- | --- |
| Amount of time a person has waited for a transplant | 1 month | 2 years |
| Tissue type match – and likelihood of transplant success. | 86% average 1 year chance of transplant success | 89% average 1 year chance of transplant success |
| **How many dependents (children or adults) recipients have.** | 1 dependents | 4 dependents |
| **Recipient age** | 45 years | 20 years |
| Diseases affecting life expectancy | Moderate: Uncontrolled hypertension or obesity | Severe: Heart attack or stroke, or diabetes with complications. |
| Other recipient illnesses (other than Kidney disease) | Severe arthritis | None |

Which patient would you choose? Patient A **❑** Patient B **❑**

(tick 1 box only)

| 5 | Patient A | Patient B |
| --- | --- | --- |
| Amount of time a person has waited for a transplant | 2 years | 10 years |
| Tissue type match – and likelihood of transplant success. | 89% average 1 year chance of transplant success | 90% average 1 year chance of transplant success |
| **How many dependents (children or adults) recipients have.** | 4 dependents | No dependents |
| **Recipient age** | 20 years | 45 years |
| Diseases affecting life expectancy | Severe: Heart attack or stroke, or diabetes with complications | None |
| Other recipient illnesses (other than Kidney disease) | None | Mild asthma |

Which patient would you choose? Patient A **❑** Patient B **❑**

(tick 1 box only)

| 6 | Patient A | Patient B |
| --- | --- | --- |
| Amount of time a person has waited for a transplant | 10 years | 1 month |
| Tissue type match – and likelihood of transplant success. | 90% average 1 year chance of transplant success | 86% average 1 year chance of transplant success |
| **How many dependents (children or adults) recipients have.** | No dependents | 1 dependent |
| **Recipient age** | 45 years | 65 years |
| Diseases affecting life expectancy | None | Moderate: Uncontrolled hypertension or obesity |
| Other recipient illnesses (other than Kidney disease) | Mild asthma | Severe arthritis |

Which patient would you choose? Patient A **❑** Patient B **❑**

(tick 1 box only)

| 7 | Patient A | Patient B |
| --- | --- | --- |
| Amount of time a person has waited for a transplant | 1 month | 2 years |
| Tissue type match – and likelihood of transplant success. | 89% average 1 year chance of transplant success | 90% average 1 year chance of transplant success |
| **How many dependents (children or adults) recipients have.** | No dependents | 1 dependent |
| **Recipient age** | 65 years | 20 years |
| Diseases affecting life expectancy | Severe: Heart attack or stroke, or diabetes with complications | None |
| Other recipient illnesses (other than Kidney disease) | Mild asthma | Severe arthritis |

Which patient would you choose? Patient A **❑** Patient B **❑**

(tick 1 box only)

| 8 | Patient A | Patient B |
| --- | --- | --- |
| Amount of time a person has waited for a transplant | 2 years | 10 years |
| Tissue type match – and likelihood of transplant success. | 90% average 1 year chance of transplant success | 86% average 1 year chance of transplant success |
| **How many dependents (children or adults) recipients have.** | 1 dependent | 4 dependents |
| **Recipient age** | 20 years | 45 years |
| Diseases affecting life expectancy | None | Moderate: Uncontrolled hypertension or obesity |
| Other recipient illnesses (other than Kidney disease) | Severe arthritis | None |

Which patient would you choose? Patient A **❑** Patient B **❑**

(tick 1 box only)

| 9 | Patient A | Patient B |
| --- | --- | --- |
| Amount of time a person has waited for a transplant | 10 years | 1 month |
| Tissue type match – and likelihood of transplant success. | 86% average 1 year chance of transplant success | 89% average 1 year chance of transplant success |
| **How many dependents (children or adults) recipients have.** | 4 dependents | No dependents |
| **Recipient age** | 45 years | 65 years |
| Diseases affecting life expectancy | Moderate: Uncontrolled hypertension or obesity | Severe: Heart attack or stroke, or diabetes with complications |
| Other recipient illnesses (other than Kidney disease) | None | Mild asthma |

Which patient would you choose? Patient A **❑** Patient B **❑**

(tick 1 box only)

**About you and your circumstances.**

**Gender:**

***(Please tick 1 box only):* Male ❑ Female ❑**

**Age: ____ years**

**Do you have any children under 18 years?**

***(Please tick 1 box only):***

**Yes ❑ No ❑**

**If ‘Yes’ how many of these live in your household?**

**1 child ❑ 2 children ❑ 3 children ❑ More than 3 children ❑**

**Do you have to care for any dependent adults *(Please tick 1 box only):***

**Yes ❑ No ❑**

**If ‘Yes’ how many?**

**1 dependent adult ❑ 2 dependent adults ❑ More than 2 dependent adults ❑**

**Which of the following ethnic groups do you consider that you belong to? *(Please tick 1 box only):***

**White – British ❑**

**White – Irish ❑**

**White – Any other white background ❑**

**- please describe__________________________**

**Mixed – White / Black Caribbean ❑**

**Mixed – White / Black African ❑**

**Mixed – White / Asian ❑**

**Any other mixed background ❑**

**- please describe__________________________**

**Black or black British (Caribbean) ❑**

**Black or black British (African) ❑**

**Black or black British (Any other background ❑**

**Asian or Asian British (Indian) ❑**

**Asian or Asian British (Pakistani) ❑**

**Asian or Asian British (Bangladeshi) ❑**

**Asian or Asian British (Any other background) ❑**

**Chinese ❑**

**Any other ethnic group ❑**

**- please describe__________________________**

**Work:**

**Are you currently in paid employment? *(Please tick all that apply):***

**Yes (Working full-time). ❑ Yes (Working part-time) ❑**

**Unemployed (Not working, ❑ Not working due to long- ❑**

**but available for work). Term sickness or disability.**

**Retired from paid work. ❑ Full time student ❑**

**Engagement in household ❑ Others *(please specify:)* ❑**

**Duties. _____________________________**

**Qualifications:**

**What is the highest level of education you have completed *(Please tick 1 box only):***

#### Secondary School ❑

**Vocational / Trade / College Qualification ❑**

**‘A’ level / ‘AS’ levels ❑**

**Degree level qualification(s) ❑**

**Other *(please describe_____________________).* ❑**

**What perspective did you adopt when answering this questionnaire?:** ***(Please tick 1 box only):***

**Answering the questions in terms of what**

#### would be best for me ❑

**Answering the questions in terms of what**

#### would be best for me and others ❑

**Disregarding what is best for me and only**

#### considering what is best for others ❑

**Are you a Transplanted patient? *(please tick all that apply)*:**

#### Currently successful.

#### Now failed – If failed how long has it been since kidney failure treatment with your first dialysis / transplant:______years______months.

**Or:**

#### On dialysis (not transplanted) – Since being on dialysis how long have you been on the transplant list?:______years______months.

#### Kidney disease but not on dialysis.

**Waiting list for transplantation *(if you are on a waiting list, or have been please complete the following as applicable):***

**Are you on the waiting list for a kidney transplant?**

**Yes ❑**

**No ❑**

**If you answered ‘yes’ how long did you wait on the list for your most recent transplant?*(if applicable)*: _______years and:_______months.**

**Are you currently on kidney dialysis? If so how long have you been on kidney dialysis?*(if applicable)*:_______years and ________months.**

**If you are no longer on kidney dialysis but have been in the past, please indicate the total amount of time you have spent on dialysis? *(if applicable)*:______years and ________months.**

#### The following questions are to ask about your general health state at the moment. By placing a tick in one box in each group below, please indicate which statement best describes your own health state today.

**Do not tick more than one box per question.**

**1. Mobility:**

I have no problems in walking about

I have some problems in walking about

I am confined to bed

**2. Self-Care:**

I have no problems with self-care

I have some problems washing or dressing myself

I am unable to wash or dress myself

**3. Usual Activities** (e.g. work, study, housework, family or leisure activities)**:**

I have no problems with performing my usual activities

I have some problems with performing my usual activities

I am unable to perform my usual activities

**4. Pain / Discomfort:**

I have no pain or discomfort

I have moderate pain or discomfort

I have extreme pain or discomfort

**5. Anxiety / Depression:**

I am not anxious or depressed

I am moderately anxious or depressed

I am extremely anxious or depressed

## Your own health state today


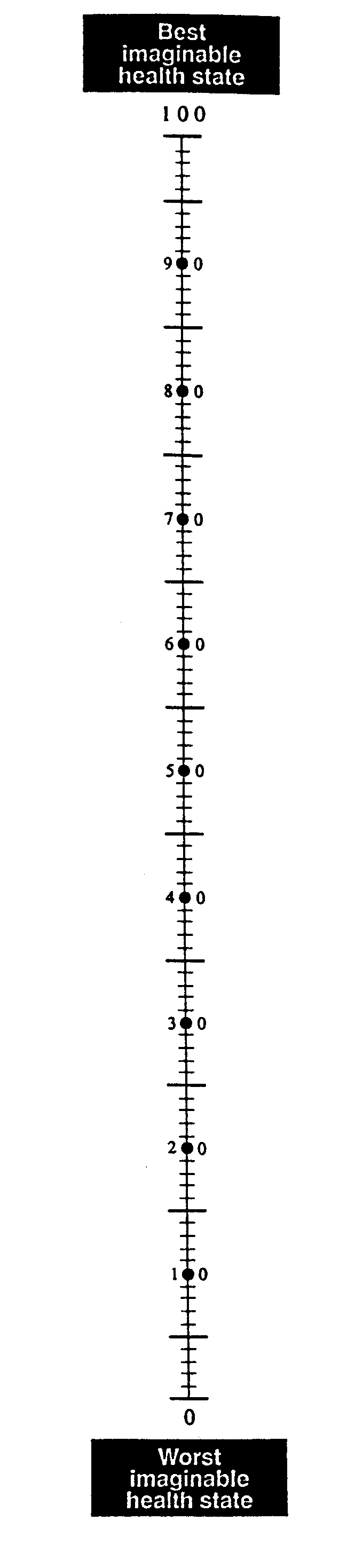


**Your own health state** **TODAY**

To help people say how good or

bad a health state is, we have drawn

a scale (rather like a thermometer)

on which the best state you can

imagine is marked by 100 and the

worst state you can imagine is

marked by 0.

We would like you to indicate on this scale **how good or bad is your own health today, in your opinion.**

Please do this by drawing a line from the box below, to whichever point on the scale indicates how good or bad your current health state is **today**.

Who should be prioritized for Kidney Transplants in the UK?

## A SURVEY OF YOUR PREFERENCES.

REF:

Undertaken by:


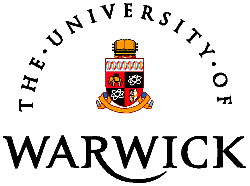


Warwick Medical School,

University of Warwick,

Coventry,

UK,

CV4 7AL.

Undertaken for:

University Hospitals of Coventry

and Warwickshire NHS Trust,

Walsgrave Hospital,

Clifford Bridge Road,

Coventry,

CV2 2DX

**Version 5b: Carer version**

# What is this questionnaire about?:

In this questionnaire we are trying to establish how to prioritize patients for a renal transplant. It is important for us to ensure that the systems for kidney allocation take account of the views of patients, health professionals and others involved in the transplant process. This questionnaire is being distributed as part of a research project aimed to provide increased information about what criteria should be used in order to allocate kidney transplants, and what weight should be attached to different criteria. This is independent research, which may in time have an impact upon kidney allocation policy. It will take into account the views of key stakeholders who may have an interest in the criteria to be used to prioritize transplants. Stakeholders will include renal patients; healthcare workers routinely working with renal patients; those caring for renal patients; kidney donors and relatives of deceased donors.

In order to do this we have developed this questionnaire. The questionnaire is trying to find out your preferences for a number of different hypothetical (illustrative) health care scenarios for treatment of patients who need a renal transplantation. Please begin by familiarising yourself with the various possible alternatives by **reading the box below.** Please assume that all other factors other than those which we indicate may differ, are equal.

Background information that we can provide you with which may inform your choices is as follows:

- This questionnaire relates only to allocation from deceased (dead) donors, currently there are over 5,000 people awaiting a transplant in the UK, and this year about 1,300 will get a deceased donor transplant.
- Under the matching system currently used in the UK, it is possible to get a kidney transplant at any time after going on the list, but someone may wait for many years and occasionally someone gets a transplant after being on the list for only a few weeks.
- The main factors used to allocate kidneys are waiting time and tissue matching. This means that people with rare or unusual tissue types have reduced chance of getting a cadaveric transplant. This applies to many people from ethnic minorities.
- A difference in tissue type between the donor and recipient was the main cause of transplant rejection in the past. However, with better anti-rejection drugs, rejection is not now the main cause of transplant loss, though a kidney transplant with an excellent tissue type now has a slightly better chance of survival than one with a reasonable match (Figures from UK Transplant: 1 year survival for perfect match it is 90%; for favourable match it is 89%; and for a non-favourable match it is 86%).

**Different criteria for prioritizing transplants**

| **Description of alternatives.**  **A) Amount of time a person has waited.**  Timescales for people receiving a transplant after being placed on a waiting list are likely to differ. The waiting time could be:  1) 1 month.  2) 2 years.  3) 10 years.  **B) Tissue type matching**  – and likelihood of transplant success.  This affects the likelihood of a transplant proving to be successful. Below are the up to date figures from UK Transplant for the survival of all transplants in the UK. There are 6 main tissue types used in matching. A perfect tissue type match is all 6 types matching; favourable is 4-5 out of 6 matching, non-favourable is less than 4 matching. If a transplant fails the patient  will return to renal dialysis.  1) Non-favourable tissue match (86% average survival rate of the kidney 1 year after the transplant).  2) Favourable tissue match (89% average survival rate of the kidney 1 year after the transplant).  3) Perfect tissue type match (90% average survival rate of the kidney 1 year after the transplant).  **C) How many dependents (either children or adults) recipients have.**  Some respondents might consider that those who have dependent children, or others who are dependent either because of their age or a physical or mental handicap, ought to be prioritized for kidney transplant. So we assume that respondents might have:  1) No dependents.  2) 1 dependent.  3) 4 dependents.  **D) Recipient age.**  The recipient could be aged either:  1) 20 years.  2) 45 years.  3) 65 years  **E) Diseases affecting life expectancy.**  As well as having kidney failure, someone may have other conditions prior to kidney transplantation which affect their life expectancy. Some of the conditions which reduce life expectancy may occur in young people, and some older people may be entirely healthy apart from kidney disease. We assume these could be either:  1) None.  2) Moderate diseases (uncontrolled hypertension or obesity).  3) Severe diseases (heart attack, or stroke, or diabetes with complications).  **F) Other recipient illnesses.**  Someone with kidney failure may have conditions other than kidney failure which are not life-threatening but do affect their quality of life. Respondents might or might not wish to allocate kidneys according to such conditions. Examples would be:-  1) Healthy except for kidney disease.  2) Kidney disease with a condition that sometimes affects their activities, such as mild asthma.  3) Kidney disease with a condition that affects their activities on a daily basis, such as severe arthritis. |
| --- |

##### INSTRUCTIONS FOR COMPLETION OF THE QUESTIONNAIRE

- We now want you to choose between different options. Please remember that the questions are hypothetical, but we want you to assume that what the questions tells you is what is actually happening.
- Everything else about the pattern of who receives what care apart from the stated differences is identical.
- Please answer every question remembering that there are no right or wrong answers, it is finding out what your personal preferences are that matters.
- Assume there is 1 kidney that could be transplanted to either patient A or patient B.

NOW PLEASE READ DESCRIPTIONS OF OPTION A AND B AND INDICATE WHO YOU THINK SHOULD BE PRIORITISED FOR A KIDNEY TRANSPLANT – PATIENT A OR PATIENT B?:

| 1 | Patient A | Patient B |
| --- | --- | --- |
| Amount of time a person has waited for a transplant | 1 month | 2 years |
| Tissue type match – and likelihood of transplant success. | 90% average 1 year chance of transplant success | 86% average 1 year chance of transplant success |
| **How many dependents (children or adults) recipients have.** | 4 dependents | No dependents |
| **Recipient age** | 20 years | 45 years |
| Diseases affecting life expectancy | Moderate: Uncontrolled hypertension or obesity | Severe: Heart attack, or stroke, or diabetes with complications. |
| Other recipient illnesses (other than Kidney disease) | Mild asthma | Severe arthritis |

Which patient would you choose? Patient A **❑** Patient B **❑**

(tick 1 box only)

| 2 | Patient A | Patient B |
| --- | --- | --- |
| Amount of time a person has waited for a transplant | 2 years | 10 years |
| Tissue type match – and likelihood of transplant success. | 86% average 1 year chance of transplant success | 89% average 1 year chance of transplant success |
| **How many dependents (children or adults) recipients have.** | No dependents | 1 dependent |
| **Recipient age** | 45 years | 65 years |
| Diseases affecting life expectancy | Severe: Heart attack, or stroke, or diabetes with complications | None |
| Other recipient illnesses (other than Kidney disease) | Severe arthritis | None |

Which patient would you choose? Patient A **❑** Patient B **❑**

(tick 1 box only)

| 3 | Patient A | Patient B |
| --- | --- | --- |
| Amount of time a person has waited for a transplant | 10 years | 1 month |
| Tissue type match – and likelihood of transplant success. | 89% average 1 year chance of transplant success | 90% average 1 year chance of transplant success |
| **How many dependents (children or adults) recipients have.** | 1 dependent | 4 dependents |
| **Recipient age** | 65 years | 20 years |
| Diseases affecting life expectancy | None | Moderate: Uncontrolled hypertension or obesity |
| Other recipient illnesses (other than Kidney disease) | None | Mild asthma |

Which patient would you choose? Patient A **❑** Patient B **❑**

(tick 1 box only)

| 4 | Patient A | Patient B |
| --- | --- | --- |
| Amount of time a person has waited for a transplant | 1 month | 2 years |
| Tissue type match – and likelihood of transplant success. | 89% average 1 year chance of transplant success | 90% average 1 year chance of transplant success |
| **How many dependents (children or adults) recipients have.** | 4 dependents | No dependents |
| **Recipient age** | 45 years | 65 years |
| Diseases affecting life expectancy | None | Moderate: Uncontrolled hypertension or obesity |
| Other recipient illnesses (other than Kidney disease) | Severe arthritis | None |

Which patient would you choose? Patient A **❑** Patient B **❑**

(tick 1 box only)

| 5 | Patient A | Patient B |
| --- | --- | --- |
| Amount of time a person has waited for a transplant | 2 years | 10 years |
| Tissue type match – and likelihood of transplant success. | 90% average 1 year chance of transplant success | 86% average 1 year chance of transplant success |
| **How many dependents (children or adults) recipients have.** | No dependents | 1 dependent |
| **Recipient age** | 65 years | 20 years |
| Diseases affecting life expectancy | Moderate: Uncontrolled hypertension or obesity | Severe: Heart attack, or stroke, or diabetes with complications. |
| Other recipient illnesses (other than Kidney disease) | None | Mild asthma |

Which patient would you choose? Patient A **❑** Patient B **❑**

(tick 1 box only)

| 6 | Patient A | Patient B |
| --- | --- | --- |
| Amount of time a person has waited for a transplant | 10 years | 1 month |
| Tissue type match – and likelihood of transplant success. | 86% average 1 year chance of transplant success | 86% average 1 year chance of transplant success |
| **How many dependents (children or adults) recipients have.** | 1 dependent | 4 dependents |
| **Recipient age** | 20 years | 45 years |
| Diseases affecting life expectancy | Severe: Heart attack, or stroke, or diabetes with complications. | None |
| Other recipient illnesses (other than Kidney disease) | Mild asthma | Severe arthritis |

Which patient would you choose? Patient A **❑** Patient B **❑**

(tick 1 box only)

| 7 | Patient A | Patient B |
| --- | --- | --- |
| Amount of time a person has waited for a transplant | 1 month | 2 years |
| Tissue type match – and likelihood of transplant success. | 90% average 1 year chance of transplant success | 86% average 1 year chance of transplant success |
| **How many dependents (children or adults) recipients have.** | 1 dependent | 4 dependents |
| **Recipient age** | 45 years | 65 years |
| Diseases affecting life expectancy | Severe: Heart attack or stroke, or diabetes with complications | None |
| Other recipient illnesses (other than Kidney disease) | None | Mild asthma |

Which patient would you choose? Patient A **❑** Patient B **❑**

(tick 1 box only)

| 8 | Patient A | Patient B |
| --- | --- | --- |
| Amount of time a person has waited for a transplant | 2 years | 10 years |
| Tissue type match – and likelihood of transplant success. | 86% average 1 year chance of transplant success | 89% average 1 year chance of transplant success |
| **How many dependents (children or adults) recipients have.** | 4 dependents | No dependents |
| **Recipient age** | 65 years | 20 years |
| Diseases affecting life expectancy | None | Moderate: Uncontrolled hypertension or obesity |
| Other recipient illnesses (other than Kidney disease) | Mild asthma | Severe arthritis |

Which patient would you choose? Patient A **❑** Patient B **❑**

(tick 1 box only)

| 9 | Patient A | Patient B |
| --- | --- | --- |
| Amount of time a person has waited for a transplant | 10 years | 1 month |
| Tissue type match – and likelihood of transplant success. | 89% average 1 year chance of transplant success | 90% average 1 year chance of transplant success |
| **How many dependents (children or adults) recipients have.** | No dependents | 1 dependent |
| **Recipient age** | 20 years | 45 years |
| Diseases affecting life expectancy | Moderate: Uncontrolled hypertension or obesity | Severe: Heart attack or stroke, or diabetes with complications |
| Other recipient illnesses (other than Kidney disease) | Severe arthritis. | None |

Which patient would you choose? Patient A **❑** Patient B **❑**

(tick 1 box only)

**About you and your circumstances.**

**Gender:**

***(Please tick 1 box only):* Male ❑ Female ❑**

**Age: ____ years**

**Do you have any children under 18 years?**

***(Please tick 1 box only):***

**Yes ❑ No ❑**

**If ‘Yes’ how many of these live in your household?**

**1 child ❑ 2 children ❑ 3 children ❑ More than 3 children ❑**

**Do you have to care for any dependent adults *(Please tick 1 box only):***

**Yes ❑ No ❑**

**If ‘Yes’ how many?**

**1 dependent adult ❑ 2 dependent adults ❑ More than 2 dependent adults ❑**

**Which of the following ethnic groups do you consider that you belong to? *(Please tick 1 box only):***

**White – British ❑**

**White – Irish ❑**

**White – Any other white background ❑**

**- please describe__________________________**

**Mixed – White / Black Caribbean ❑**

**Mixed – White / Black African ❑**

**Mixed – White / Asian ❑**

**Any other mixed background ❑**

**- please describe__________________________**

**Black or black British (Caribbean) ❑**

**Black or black British (African) ❑**

**Black or black British (Any other background ❑**

**Asian or Asian British (Indian) ❑**

**Asian or Asian British (Pakistani) ❑**

**Asian or Asian British (Bangladeshi) ❑**

**Asian or Asian British (Any other background) ❑**

**Chinese ❑**

**Any other ethnic group ❑**

**- please describe__________________________**

**Work:**

**Are you currently in paid employment? *(Please tick all that apply):***

**Yes (Working full-time). ❑ Yes (Working part-time) ❑**

**Unemployed (Not working, ❑ Not working due to long- ❑**

**but available for work). Term sickness or disability.**

**Retired from paid work. ❑ Full time student ❑**

**Engagement in household ❑ Others *(please specify:)* ❑**

**Duties. _____________________________**

**Qualifications:**

**What is the highest level of education you have completed *(Please tick 1 box only):***

#### Secondary School ❑

**Vocational / Trade / College Qualification ❑**

**‘A’ level / ‘AS’ levels ❑**

**Degree level qualification(s) ❑**

**Other *(please describe_____________________).* ❑**

**What perspective did you adopt when answering this questionnaire?:** ***(Please tick 1 box only):***

**Answering the questions in terms of what**

#### would be best for me ❑

**Answering the questions in terms of what**

#### would be best for me and others ❑

**Disregarding what is best for me and only**

#### considering what is best for others ❑

**Which of the following categories does the person you are caring for fall into:**

**The person I am caring for is a transplanted patient *(please tick all that apply)*:**

#### Currently successful.

#### Now failed – If failed how long has it been since kidney failure treatment with your first dialysis / transplant:______years______months.

**Or:**

#### On dialysis – Since being on dialysis how long have they been on the transplant list?:______years______months.

#### Kidney disease but not on dialysis.

**Is the person you are caring for on the waiting list for transplantation?**

**Yes ❑**

**No ❑**

**If you answered ‘yes’ how long did she / he wait on the list for their most recent transplant?*(if applicable)*: _______years and:_______months.**

**Are they currently on kidney dialysis? If so how long have they been on kidney dialysis?*(if applicable)*:_______years and ________months.**

**If they are no longer on kidney dialysis but have been in the past, please indicate the total amount of time they have spent on dialysis? *(if applicable)*:______years and ________months.**

Who should be prioritized for Kidney Transplants in the UK?

## A SURVEY OF YOUR PREFERENCES.

REF:

Undertaken by:


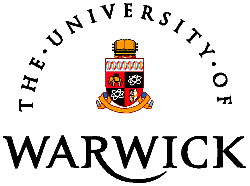


Warwick Medical School,

University of Warwick,

Coventry,

UK,

CV4 7AL.

Undertaken for:

University Hospitals of Coventry

and Warwickshire NHS Trust,

Walsgrave Hospital,

Clifford Bridge Road,

Coventry,

CV2 2DX

**Version 6a: Healthcare professional version**

# What is this questionnaire about?:

In this questionnaire we are trying to establish how to prioritize patients for a renal transplant. It is important for us to ensure that the systems for kidney allocation take account of the views of patients, health professionals and others involved in the transplant process. This questionnaire is being distributed as part of a research project aimed to provide increased information about what criteria should be used in order to allocate kidney transplants, and what weight should be attached to different criteria. This is independent research, which may in time have an impact upon kidney allocation policy. It will take into account the views of key stakeholders who may have an interest in the criteria to be used to prioritize transplants. Stakeholders will include renal patients; healthcare workers routinely working with renal patients; those caring for renal patients; kidney donors and relatives of deceased donors.

In order to do this we have developed this questionnaire. The questionnaire is trying to find out your preferences for a number of different hypothetical (illustrative) health care scenarios for treatment of patients who need a renal transplantation. Please begin by familiarising yourself with the various possible alternatives by **reading the box below.** Please assume that all other factors other than those which we indicate may differ, are equal.

Background information that we can provide you with which may inform your choices is as follows:

- This questionnaire relates only to allocation from deceased (dead) donors, currently there are over 5,000 people awaiting a transplant in the UK, and this year about 1,300 will get a deceased donor transplant.
- Under the matching system currently used in the UK, it is possible to get a kidney transplant at any time after going on the list, but someone may wait for many years and occasionally someone gets a transplant after being on the list for only a few weeks.
- The main factors used to allocate kidneys are waiting time and tissue matching. This means that people with rare or unusual tissue types have reduced chance of getting a cadaveric transplant. This applies to many people from ethnic minorities.
- A difference in tissue type between the donor and recipient was the main cause of transplant rejection in the past. However, with better anti-rejection drugs, rejection is not now the main cause of transplant loss, though a kidney transplant with an excellent tissue type now has a slightly better chance of survival than one with a reasonable match (Figures from UK Transplant: 1 year survival for perfect match it is 90%; for favourable match it is 89%; and for a non-favourable match it is 86%).

**Different criteria for prioritizing transplants**

| **Description of alternatives.**  **A) Amount of time a person has waited.**  Timescales for people receiving a transplant after being placed on a waiting list are likely to differ. The waiting time could be:  1) 1 month.  2) 2 years.  3) 10 years.  **B) Tissue type matching**  – and likelihood of transplant success.  This affects the likelihood of a transplant proving to be successful. Below are the up to date figures from UK Transplant for the survival of all transplants in the UK. There are 6 main tissue types used in matching. A perfect tissue type match is all 6 types matching; favourable is 4-5 out of 6 matching, non-favourable is less than 4 matching. If a transplant fails the patient  will return to renal dialysis.  1) Non-favourable tissue match (86% average survival rate of the kidney 1 year after the transplant).  2) Favourable tissue match (89% average survival rate of the kidney 1 year after the transplant).  3) Perfect tissue type match (90% average survival rate of the kidney 1 year after the transplant).  **C) How many dependents (either children or adults) recipients have.**  Some respondents might consider that those who have dependent children, or others who are dependent either because of their age or a physical or mental handicap, ought to be prioritized for kidney transplant. So we assume that respondents might have:  1) No dependents.  2) 1 dependent.  3) 4 dependents.  **D) Recipient age.**  The recipient could be aged either:  1) 20 years.  2) 45 years.  3) 65 years  **E) Diseases affecting life expectancy.**  As well as having kidney failure, someone may have other conditions prior to kidney transplantation which affect their life expectancy. Some of the conditions which reduce life expectancy may occur in young people, and some older people may be entirely healthy apart from kidney disease. We assume these could be either:  1) None.  2) Moderate diseases (uncontrolled hypertension or obesity).  3) Severe diseases (heart attack, or stroke, or diabetes with complications).  **F) Other recipient illnesses.**  Someone with kidney failure may have conditions other than kidney failure which are not life-threatening but do affect their quality of life. Respondents might or might not wish to allocate kidneys according to such conditions. Examples would be:-  1) Healthy except for kidney disease.  2) Kidney disease with a condition that sometimes affects their activities, such as mild asthma.  3) Kidney disease with a condition that affects their activities on a daily basis, such as severe arthritis. |
| --- |

##### INSTRUCTIONS FOR COMPLETION OF THE QUESTIONNAIRE

- We now want you to choose between different options. Please remember that the questions are hypothetical, but we want you to assume that what the questions tells you is what is actually happening.
- Everything else about the pattern of who receives what care apart from the stated differences is identical.
- Please answer every question remembering that there are no right or wrong answers, it is finding out what your personal preferences are that matters.
- Assume there is 1 kidney that could be transplanted to either patient A or patient B.

NOW PLEASE READ DESCRIPTIONS OF OPTION A AND B AND INDICATE WHO YOU THINK SHOULD BE PRIORITISED FOR A KIDNEY TRANSPLANT – PATIENT A OR PATIENT B?:

| 1 | Patient A | Patient B |
| --- | --- | --- |
| Amount of time a person has waited for a transplant | 1 month | 2 years |
| Tissue type match – and likelihood of transplant success. | 86% average 1 year chance of transplant success | 89% average 1 year chance of transplant success |
| **How many dependents (children or adults) recipients have.** | No dependents | 1 dependent |
| **Recipient age** | 20 years | 45 years |
| Diseases affecting life expectancy | None | Moderate: Uncontrolled hypertension or obesity |
| Other recipient illnesses (other than Kidney disease) | None | Mild asthma |

Which patient would you choose? Patient A **❑** Patient B **❑**

(tick 1 box only)

| 2 | Patient A | Patient B |
| --- | --- | --- |
| Amount of time a person has waited for a transplant | 2 years | 10 years |
| Tissue type match – and likelihood of transplant success. | 89% average 1 year chance of transplant success | 90% average 1 year chance of transplant success |
| **How many dependents (children or adults) recipients have.** | 1 dependents | 4 dependents |
| **Recipient age** | 45 years | 65 years |
| Diseases affecting life expectancy | Moderate: Uncontrolled hypertension or obesity | Severe: Heart attack, or stroke, or diabetes with complications. |
| Other recipient illnesses (other than Kidney disease) | Mild asthma | Severe arthritis |

Which patient would you choose? Patient A **❑** Patient B **❑**

(tick 1 box only)

| 3 | Patient A | Patient B |
| --- | --- | --- |
| Amount of time a person has waited for a transplant | 10 years | 1 month |
| Tissue type match – and likelihood of transplant success. | 90% average 1 year chance of transplant success | 86% average 1 year chance of transplant success |
| **How many dependents (children or adults) recipients have.** | 4 dependents | No dependents |
| **Recipient age** | 65 years | 20 years |
| Diseases affecting life expectancy | Severe: Heart attack, or stroke, or diabetes with complications | None |
| Other recipient illnesses (other than Kidney disease) | Severe arthritis | None |

Which patient would you choose? Patient A **❑** Patient B **❑**

(tick 1 box only)

| 4 | Patient A | Patient B |
| --- | --- | --- |
| Amount of time a person has waited for a transplant | 1 month | 2 years |
| Tissue type match – and likelihood of transplant success. | 86% average 1 year chance of transplant success | 89% average 1 year chance of transplant success |
| **How many dependents (children or adults) recipients have.** | 1 dependents | 4 dependents |
| **Recipient age** | 45 years | 20 years |
| Diseases affecting life expectancy | Moderate: Uncontrolled hypertension or obesity | Severe: Heart attack or stroke, or diabetes with complications. |
| Other recipient illnesses (other than Kidney disease) | Severe arthritis | None |

Which patient would you choose? Patient A **❑** Patient B **❑**

(tick 1 box only)

| 5 | Patient A | Patient B |
| --- | --- | --- |
| Amount of time a person has waited for a transplant | 2 years | 10 years |
| Tissue type match – and likelihood of transplant success. | 89% average 1 year chance of transplant success | 90% average 1 year chance of transplant success |
| **How many dependents (children or adults) recipients have.** | 4 dependents | No dependents |
| **Recipient age** | 20 years | 45 years |
| Diseases affecting life expectancy | Severe: Heart attack or stroke, or diabetes with complications | None |
| Other recipient illnesses (other than Kidney disease) | None | Mild asthma |

Which patient would you choose? Patient A **❑** Patient B **❑**

(tick 1 box only)

| 6 | Patient A | Patient B |
| --- | --- | --- |
| Amount of time a person has waited for a transplant | 10 years | 1 month |
| Tissue type match – and likelihood of transplant success. | 90% average 1 year chance of transplant success | 86% average 1 year chance of transplant success |
| **How many dependents (children or adults) recipients have.** | No dependents | 1 dependent |
| **Recipient age** | 45 years | 65 years |
| Diseases affecting life expectancy | None | Moderate: Uncontrolled hypertension or obesity |
| Other recipient illnesses (other than Kidney disease) | Mild asthma | Severe arthritis |

Which patient would you choose? Patient A **❑** Patient B **❑**

(tick 1 box only)

| 7 | Patient A | Patient B |
| --- | --- | --- |
| Amount of time a person has waited for a transplant | 1 month | 2 years |
| Tissue type match – and likelihood of transplant success. | 89% average 1 year chance of transplant success | 90% average 1 year chance of transplant success |
| **How many dependents (children or adults) recipients have.** | No dependents | 1 dependent |
| **Recipient age** | 65 years | 20 years |
| Diseases affecting life expectancy | Severe: Heart attack or stroke, or diabetes with complications | None |
| Other recipient illnesses (other than Kidney disease) | Mild asthma | Severe arthritis |

Which patient would you choose? Patient A **❑** Patient B **❑**

(tick 1 box only)

| 8 | Patient A | Patient B |
| --- | --- | --- |
| Amount of time a person has waited for a transplant | 2 years | 10 years |
| Tissue type match – and likelihood of transplant success. | 90% average 1 year chance of transplant success | 86% average 1 year chance of transplant success |
| **How many dependents (children or adults) recipients have.** | 1 dependent | 4 dependents |
| **Recipient age** | 20 years | 45 years |
| Diseases affecting life expectancy | None | Moderate: Uncontrolled hypertension or obesity |
| Other recipient illnesses (other than Kidney disease) | Severe arthritis | None |

Which patient would you choose? Patient A **❑** Patient B **❑**

(tick 1 box only)

| 9 | Patient A | Patient B |
| --- | --- | --- |
| Amount of time a person has waited for a transplant | 10 years | 1 month |
| Tissue type match – and likelihood of transplant success. | 86% average 1 year chance of transplant success | 89% average 1 year chance of transplant success |
| **How many dependents (children or adults) recipients have.** | 4 dependents | No dependents |
| **Recipient age** | 45 years | 65 years |
| Diseases affecting life expectancy | Moderate: Uncontrolled hypertension or obesity | Severe: Heart attack or stroke, or diabetes with complications |
| Other recipient illnesses (other than Kidney disease) | None | Mild asthma |

Which patient would you choose? Patient A **❑** Patient B **❑**

(tick 1 box only)

**About you and your circumstances.**

**Gender:**

***(Please tick 1 box only):* Male ❑ Female ❑**

**Age: ____ years**

**Do you have any children under 18 years?**

***(Please tick 1 box only):***

**Yes ❑ No ❑**

**If ‘Yes’ how many of these live in your household?**

**1 child ❑ 2 children ❑ 3 children ❑ More than 3 children ❑**

**Do you have to care for any dependent adults *(Please tick 1 box only):***

**Yes ❑ No ❑**

**If ‘Yes’ how many?**

**1 dependent adult ❑ 2 dependent adults ❑ More than 2 dependent adults ❑**

**Which of the following ethnic groups do you consider that you belong to? *(Please tick 1 box only):***

**White – British ❑**

**White – Irish ❑**

**White – Any other white background ❑**

**- please describe__________________________**

**Mixed – White / Black Caribbean ❑**

**Mixed – White / Black African ❑**

**Mixed – White / Asian ❑**

**Any other mixed background ❑**

**- please describe__________________________**

**Black or black British (Caribbean) ❑**

**Black or black British (African) ❑**

**Black or black British (Any other background ❑**

**Asian or Asian British (Indian) ❑**

**Asian or Asian British (Pakistani) ❑**

**Asian or Asian British (Bangladeshi) ❑**

**Asian or Asian British (Any other background) ❑**

**Chinese ❑**

**Any other ethnic group ❑**

**- please describe__________________________**

**Work:**

**Are you currently in paid employment? *(Please tick all that apply):***

**Yes (Working full-time). ❑ Yes (Working part-time) ❑**

**Unemployed (Not working, ❑ Not working due to long- ❑**

**but available for work). Term sickness or disability.**

**Retired from paid work. ❑ Full time student ❑**

**Engagement in household ❑ Others *(please specify:)* ❑**

**Duties. _____________________________**

**Qualifications:**

**What is the highest level of education you have completed *(Please tick 1 box only):***

#### Secondary School ❑

**Vocational / Trade / College Qualification ❑**

**‘A’ level / ‘AS’ levels ❑**

**Degree level qualification(s) ❑**

**Other *(please describe_____________________).* ❑**

**What perspective did you adopt when answering this questionnaire?:** ***(Please tick 1 box only):***

**Answering the questions in terms of what**

#### would be best for me ❑

**Answering the questions in terms of what**

#### would be best for me and others ❑

**Disregarding what is best for me and only**

#### considering what is best for others ❑

Which of the following categories do you fall into:

#### Surgeon.

####

#### Renal Physician.

####

#### Transplant co-ordinator.

####

#### Nurse.

####

#### Pharmacist.

**❑ Other *(please describe)_________________________________________.***

Who should be prioritized for Kidney Transplants in the UK?

## A SURVEY OF YOUR PREFERENCES.

REF:

Undertaken by:


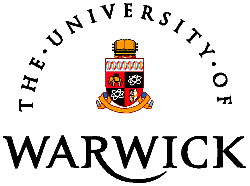


Warwick Medical School,

University of Warwick,

Coventry,

UK,

CV4 7AL.

Undertaken for:

University Hospitals of Coventry

and Warwickshire NHS Trust,

Walsgrave Hospital,

Clifford Bridge Road,

Coventry,

CV2 2DX

**Version 7b: Donor / relative of deceased donor version**

# What is this questionnaire about?:

In this questionnaire we are trying to establish how to prioritize patients for a renal transplant. It is important for us to ensure that the systems for kidney allocation take account of the views of patients, health professionals and others involved in the transplant process. This questionnaire is being distributed as part of a research project aimed to provide increased information about what criteria should be used in order to allocate kidney transplants, and what weight should be attached to different criteria. This is independent research, which may in time have an impact upon kidney allocation policy. It will take into account the views of key stakeholders who may have an interest in the criteria to be used to prioritize transplants. Stakeholders will include renal patients; healthcare workers routinely working with renal patients; those caring for renal patients; kidney donors and relatives of deceased donors.

In order to do this we have developed this questionnaire. The questionnaire is trying to find out your preferences for a number of different hypothetical (illustrative) health care scenarios for treatment of patients who need a renal transplantation. Please begin by familiarising yourself with the various possible alternatives by **reading the box below.** Please assume that all other factors other than those which we indicate may differ, are equal.

Background information that we can provide you with which may inform your choices is as follows:

- This questionnaire relates only to allocation from deceased (dead) donors, currently there are over 5,000 people awaiting a transplant in the UK, and this year about 1,300 will get a deceased donor transplant.
- Under the matching system currently used in the UK, it is possible to get a kidney transplant at any time after going on the list, but someone may wait for many years and occasionally someone gets a transplant after being on the list for only a few weeks.
- The main factors used to allocate kidneys are waiting time and tissue matching. This means that people with rare or unusual tissue types have reduced chance of getting a cadaveric transplant. This applies to many people from ethnic minorities.
- A difference in tissue type between the donor and recipient was the main cause of transplant rejection in the past. However, with better anti-rejection drugs, rejection is not now the main cause of transplant loss, though a kidney transplant with an excellent tissue type now has a slightly better chance of survival than one with a reasonable match (Figures from UK Transplant: 1 year survival for perfect match it is 90%; for favourable match it is 89%; and for a non-favourable match it is 86%).

**Different criteria for prioritizing transplants**

| **Description of alternatives.**  **A) Amount of time a person has waited.**  Timescales for people receiving a transplant after being placed on a waiting list are likely to differ. The waiting time could be:  1) 1 month.  2) 2 years.  3) 10 years.  **B) Tissue type matching**  – and likelihood of transplant success.  This affects the likelihood of a transplant proving to be successful. Below are the up to date figures from UK Transplant for the survival of all transplants in the UK. There are 6 main tissue types used in matching. A perfect tissue type match is all 6 types matching; favourable is 4-5 out of 6 matching, non-favourable is less than 4 matching. If a transplant fails the patient  will return to renal dialysis.  1) Non-favourable tissue match (86% average survival rate of the kidney 1 year after the transplant).  2) Favourable tissue match (89% average survival rate of the kidney 1 year after the transplant).  3) Perfect tissue type match (90% average survival rate of the kidney 1 year after the transplant).  **C) How many dependents (either children or adults) recipients have.**  Some respondents might consider that those who have dependent children, or others who are dependent either because of their age or a physical or mental handicap, ought to be prioritized for kidney transplant. So we assume that respondents might have:  1) No dependents.  2) 1 dependent.  3) 4 dependents.  **D) Recipient age.**  The recipient could be aged either:  1) 20 years.  2) 45 years.  3) 65 years  **E) Diseases affecting life expectancy.**  As well as having kidney failure, someone may have other conditions prior to kidney transplantation which affect their life expectancy. Some of the conditions which reduce life expectancy may occur in young people, and some older people may be entirely healthy apart from kidney disease. We assume these could be either:  1) None.  2) Moderate diseases (uncontrolled hypertension or obesity).  3) Severe diseases (heart attack, or stroke, or diabetes with complications).  **F) Other recipient illnesses.**  Someone with kidney failure may have conditions other than kidney failure which are not life-threatening but do affect their quality of life. Respondents might or might not wish to allocate kidneys according to such conditions. Examples would be:-  1) Healthy except for kidney disease.  2) Kidney disease with a condition that sometimes affects their activities, such as mild asthma.  3) Kidney disease with a condition that affects their activities on a daily basis, such as severe arthritis. |
| --- |

##### INSTRUCTIONS FOR COMPLETION OF THE QUESTIONNAIRE

- We now want you to choose between different options. Please remember that the questions are hypothetical, but we want you to assume that what the questions tells you is what is actually happening.
- Everything else about the pattern of who receives what care apart from the stated differences is identical.
- Please answer every question remembering that there are no right or wrong answers, it is finding out what your personal preferences are that matters.
- Assume there is 1 kidney that could be transplanted to either patient A or patient B.

NOW PLEASE READ DESCRIPTIONS OF OPTION A AND B AND INDICATE WHO YOU THINK SHOULD BE PRIORITISED FOR A KIDNEY TRANSPLANT – PATIENT A OR PATIENT B?:

| 1 | Patient A | Patient B |
| --- | --- | --- |
| Amount of time a person has waited for a transplant | 1 month | 2 years |
| Tissue type match – and likelihood of transplant success. | 90% average 1 year chance of transplant success | 86% average 1 year chance of transplant success |
| **How many dependents (children or adults) recipients have.** | 4 dependents | No dependents |
| **Recipient age** | 20 years | 45 years |
| Diseases affecting life expectancy | Moderate: Uncontrolled hypertension or obesity | Severe: Heart attack, or stroke, or diabetes with complications. |
| Other recipient illnesses (other than Kidney disease) | Mild asthma | Severe arthritis |

Which patient would you choose? Patient A **❑** Patient B **❑**

(tick 1 box only)

| 2 | Patient A | Patient B |
| --- | --- | --- |
| Amount of time a person has waited for a transplant | 2 years | 10 years |
| Tissue type match – and likelihood of transplant success. | 86% average 1 year chance of transplant success | 89% average 1 year chance of transplant success |
| **How many dependents (children or adults) recipients have.** | No dependents | 1 dependent |
| **Recipient age** | 45 years | 65 years |
| Diseases affecting life expectancy | Severe: Heart attack, or stroke, or diabetes with complications | None |
| Other recipient illnesses (other than Kidney disease) | Severe arthritis | None |

Which patient would you choose? Patient A **❑** Patient B **❑**

(tick 1 box only)

| 3 | Patient A | Patient B |
| --- | --- | --- |
| Amount of time a person has waited for a transplant | 10 years | 1 month |
| Tissue type match – and likelihood of transplant success. | 89% average 1 year chance of transplant success | 90% average 1 year chance of transplant success |
| **How many dependents (children or adults) recipients have.** | 1 dependent | 4 dependents |
| **Recipient age** | 65 years | 20 years |
| Diseases affecting life expectancy | None | Moderate: Uncontrolled hypertension or obesity |
| Other recipient illnesses (other than Kidney disease) | None | Mild asthma |

Which patient would you choose? Patient A **❑** Patient B **❑**

(tick 1 box only)

| 4 | Patient A | Patient B |
| --- | --- | --- |
| Amount of time a person has waited for a transplant | 1 month | 2 years |
| Tissue type match – and likelihood of transplant success. | 89% average 1 year chance of transplant success | 90% average 1 year chance of transplant success |
| **How many dependents (children or adults) recipients have.** | 4 dependents | No dependents |
| **Recipient age** | 45 years | 65 years |
| Diseases affecting life expectancy | None | Moderate: Uncontrolled hypertension or obesity |
| Other recipient illnesses (other than Kidney disease) | Severe arthritis | None |

Which patient would you choose? Patient A **❑** Patient B **❑**

(tick 1 box only)

| 5 | Patient A | Patient B |
| --- | --- | --- |
| Amount of time a person has waited for a transplant | 2 years | 10 years |
| Tissue type match – and likelihood of transplant success. | 90% average 1 year chance of transplant success | 86% average 1 year chance of transplant success |
| **How many dependents (children or adults) recipients have.** | No dependents | 1 dependent |
| **Recipient age** | 65 years | 20 years |
| Diseases affecting life expectancy | Moderate: Uncontrolled hypertension or obesity | Severe: Heart attack, or stroke, or diabetes with complications. |
| Other recipient illnesses (other than Kidney disease) | None | Mild asthma |

Which patient would you choose? Patient A **❑** Patient B **❑**

(tick 1 box only)

| 6 | Patient A | Patient B |
| --- | --- | --- |
| Amount of time a person has waited for a transplant | 10 years | 1 month |
| Tissue type match – and likelihood of transplant success. | 86% average 1 year chance of transplant success | 86% average 1 year chance of transplant success |
| **How many dependents (children or adults) recipients have.** | 1 dependent | 4 dependents |
| **Recipient age** | 20 years | 45 years |
| Diseases affecting life expectancy | Severe: Heart attack, or stroke, or diabetes with complications. | None |
| Other recipient illnesses (other than Kidney disease) | Mild asthma | Severe arthritis |

Which patient would you choose? Patient A **❑** Patient B **❑**

(tick 1 box only)

| 7 | Patient A | Patient B |
| --- | --- | --- |
| Amount of time a person has waited for a transplant | 1 month | 2 years |
| Tissue type match – and likelihood of transplant success. | 90% average 1 year chance of transplant success | 86% average 1 year chance of transplant success |
| **How many dependents (children or adults) recipients have.** | 1 dependent | 4 dependents |
| **Recipient age** | 45 years | 65 years |
| Diseases affecting life expectancy | Severe: Heart attack or stroke, or diabetes with complications | None |
| Other recipient illnesses (other than Kidney disease) | None | Mild asthma |

Which patient would you choose? Patient A **❑** Patient B **❑**

(tick 1 box only)

| 8 | Patient A | Patient B |
| --- | --- | --- |
| Amount of time a person has waited for a transplant | 2 years | 10 years |
| Tissue type match – and likelihood of transplant success. | 86% average 1 year chance of transplant success | 89% average 1 year chance of transplant success |
| **How many dependents (children or adults) recipients have.** | 4 dependents | No dependents |
| **Recipient age** | 65 years | 20 years |
| Diseases affecting life expectancy | None | Moderate: Uncontrolled hypertension or obesity |
| Other recipient illnesses (other than Kidney disease) | Mild asthma | Severe arthritis |

Which patient would you choose? Patient A **❑** Patient B **❑**

(tick 1 box only)

| 9 | Patient A | Patient B |
| --- | --- | --- |
| Amount of time a person has waited for a transplant | 10 years | 1 month |
| Tissue type match – and likelihood of transplant success. | 89% average 1 year chance of transplant success | 90% average 1 year chance of transplant success |
| **How many dependents (children or adults) recipients have.** | No dependents | 1 dependent |
| **Recipient age** | 20 years | 45 years |
| Diseases affecting life expectancy | Moderate: Uncontrolled hypertension or obesity | Severe: Heart attack or stroke, or diabetes with complications |
| Other recipient illnesses (other than Kidney disease) | Severe arthritis. | None |

Which patient would you choose? Patient A **❑** Patient B **❑**

(tick 1 box only)

**About you and your circumstances.**

**Gender:**

***(Please tick 1 box only):* Male ❑ Female ❑**

**Age: ____ years**

**Do you have any children under 18 years?**

***(Please tick 1 box only):***

**Yes ❑ No ❑**

**If ‘Yes’ how many of these live in your household?**

**1 child ❑ 2 children ❑ 3 children ❑ More than 3 children ❑**

**Do you have to care for any dependent adults *(Please tick 1 box only):***

**Yes ❑ No ❑**

**If ‘Yes’ how many?**

**1 dependent adult ❑ 2 dependent adults ❑ More than 2 dependent adults ❑**

**Which of the following ethnic groups do you consider that you belong to? *(Please tick 1 box only):***

**White – British ❑**

**White – Irish ❑**

**White – Any other white background ❑**

**- please describe__________________________**

**Mixed – White / Black Caribbean ❑**

**Mixed – White / Black African ❑**

**Mixed – White / Asian ❑**

**Any other mixed background ❑**

**- please describe__________________________**

**Black or black British (Caribbean) ❑**

**Black or black British (African) ❑**

**Black or black British (Any other background ❑**

**Asian or Asian British (Indian) ❑**

**Asian or Asian British (Pakistani) ❑**

**Asian or Asian British (Bangladeshi) ❑**

**Asian or Asian British (Any other background) ❑**

**Chinese ❑**

**Any other ethnic group ❑**

**- please describe__________________________**

**Work:**

**Are you currently in paid employment? *(Please tick all that apply):***

**Yes (Working full-time). ❑ Yes (Working part-time) ❑**

**Unemployed (Not working, ❑ Not working due to long- ❑**

**but available for work). Term sickness or disability.**

**Retired from paid work. ❑ Full time student ❑**

**Engagement in household ❑ Others *(please specify:)* ❑**

**Duties. _____________________________**

**Qualifications:**

**What is the highest level of education you have completed *(Please tick 1 box only):***

#### Secondary School ❑

**Vocational / Trade / College Qualification ❑**

**‘A’ level / ‘AS’ levels ❑**

**Degree level qualification(s) ❑**

**Other *(please describe_____________________).* ❑**

**What perspective did you adopt when answering this questionnaire?:** ***(Please tick 1 box only):***

**Answering the questions in terms of what**

#### would be best for me ❑

**Answering the questions in terms of what**

#### would be best for me and others ❑

**Disregarding what is best for me and only**

#### considering what is best for others ❑

**Are you a donor or relative of a deceased donor?:**

#### A living donor.

#### A relative of a deceased donor.
