# Supplementary material for: Who should be prioritized for renal transplantation?: Analysis of key stakeholder preferences using discrete choice experiments
Source: BMC Nephrol. 2012 Nov 22;13:152. doi: 10.1186/1471-2369-13-152 (PMC3576250; doi:10.1186/1471-2369-13-152)
Supplement: Additional file 3 — Technical details about the econometric models (Model 1 and Model 2) used to establish stakeholder preferences ‘Econometric / statistical analysis.’ Description: This file provides a more detailed description of the econometric models used to underpin point 6 of the ‘Methods’ section. [file 1471-2369-13-152-S3.doc]

# Additional files

**Additional file 3 – Technical details about the econometric models (Model 1 and Model 2) used to establish stakeholder preferences**

We used Random Effects Probit (Model 1), to establish stakeholder preferences.

Y_ij_ = β_0_ + β_1_wait_ij_ +β_2_tiss_ij_ +β_3_dep_ij_+β_4_age_ij_+β_5_dis1_ij_ +β_6_dis2_ij_ +β_7_ill1_ij_+β_8_ill2_ij_ + β_9_D_cij_+

β_10_D_cij_wait_ij_ +β_11_D_cij_tiss_ij_ +β_12ij_D_c_dep_ij_ +β_13ij_D_c_age_ij_ +β_14_D_cij_dis1_ij_ +β_15_D_cij_dis2_ij_ +β_16_D_cij_ill1_ij_

+β_17_D_cij_ill2_ij_+β_18_D_dij_+β_19_D_dij_wait_ij_+β_20_D_dij_tiss_ij_+β_21_D_dij_dep_ij_+β_22_D_dij_age_ij_+β_23_D_dij_dis1_ij_

+β_24_D_dij_dis2_ij_+β_25_D_dij_ill1_ij_+β_26_D_ijd_ill2_ij_+β_27_D_hij_+β_28_D_hij_wait_ij_+β_29_D_hij_tiss_ij_+β_30_D_hij_dep_ij_+

β_31_D_hij_age_ij_+β_32_D_hij_dis1_ij_ +β_33_D_hij_dis2_ij_+β_34_D_hij_ill1_ij_+β_35_D_hij_ill2_ij_+µ_i_+ξ_ij_

(Model 1)

The term Y_ij_ is a binary dependent variable, from individuals i = 1...m, for observations j = 1...n_i_. Observations n_i_ vary because the i individuals do not all complete every pairwise choice (some respondents do not answer all choices), µ_i_ is the random effects error term (which allows for multiple responses from i respondents), and ξ_ij_ is the probit error term for individuals i for j observations.

Variables are defined in table i in the manuscript. Prefixes on dummy variables (D_c_, D_d_, and D_h_), indicate carer, donor, and healthcare professional preferences respectively, D_c_ = 1 for carers, 0 otherwise; D_d_ = 1 for donor / relatives of deceased donors, 0 otherwise; and D_h_ = 1 for healthcare professionals, 0 otherwise.

Model 1 establishes whether carers, donors, or healthcare professional preferences differ from patients. If β_9_, β_18_, or β_27_, are significant this indicates general non-attribute specific differences in carer, donor, or healthcare professional preferences compared to patients. Other dummy variables are interaction dummies. If any carer coefficients (β_10…._β_17_) are significant, this indicates that preferences for associated attribute(s) differ between carers and patients. If dummies for donors (β_19…._β_26_) are significant, it indicates preferences between donors and patients for the attribute(s) differ. Likewise, if interaction dummies for healthcare workers (β_28…._β_35_) are significant, it indicates different preferences between them and patients for attribute(s).

Model 2 compares ethnic and non-ethnic minority patient preferences. The ethnic minority patient category included all patients in an ethnic category except ‘White British.’ Y_ij_, μ_i_, and ξ_ij_ are as previously defined, D_E_ is a dummy variable, D_E_ = 1, for ethnic minorities, 0 otherwise.

Y_ij_ = β_0_ +β_1_wait_ij_+β_2_tiss_ij_+β_3_dep_ij_+β_4_age_ij_+β_5_dis1_ij_+β_6_dis2_ij_+β_7_ill1_ij_+β_8_ill2_ij_+β_9_D_Eij_+β_10_D_Eij_wait_ij_

+β_11_D_Eij_tiss_ij_+β_12_D_Eij_dep_ij_+β_13_D_Eij_age_ij_+β_14_D_Eij_dis1_ij_+β_15_D_Eij_dis1_ij_+β_16_D_Eij_ill1_ij_+β_17_D_Eij_ill2_ij_+µ_i_+

ξ_ij_

(Model 2)

Model 2 establishes whether preferences differ between ethnic and non-ethnic minority patients. If β_9_ is significant it suggests non-attribute specific differences in preferences between ethnic and non-ethnic minority patients. If ethnic minority interaction dummies (β_10…._β_17_) are significant, it indicates preferences differ between ethnic minorities and non-ethnic minorities for significant associated attribute(s).
