# Supplementary material for: Who should be prioritized for renal transplantation?: Analysis of key stakeholder preferences using discrete choice experiments
Source: BMC Nephrol. 2012 Nov 22;13:152. doi: 10.1186/1471-2369-13-152 (PMC3576250; doi:10.1186/1471-2369-13-152)
Supplement: Additional file 4 — Deriving Marginal Rate of Substitution (MRS) for attributes with respect to waiting time. Description: This file provides information on how marginal rates of substitution (point 7 of the ‘Methods’ section) were calculated using data obtained from econometric models 1 and 2 (outlined in Additional file 3). [file 1471-2369-13-152-S4.doc]

# Additional files

**Additional file 4 – Deriving Marginal Rate of Substitution (MRS) for attributes with respect to waiting time.**

This table should be read in conjunction with **Additional file 3**, which provides information on the underlying equations relating to the variables outlined below.

**Table 1: Calculating MRS – Valuing attributes compared to a 1 year difference in waiting time**

| **Model 1**  **Variable** | **Patient MRS** | **Model 1**  **Variable** | **Carer MRS** |
| --- | --- | --- | --- |
| **Wait** |  | **Wait** |  |
| **Tiss** | β_2_ / β_1_ | **Tiss** | (β_2_ + β_11_) / (β_1_ + β_10_) |
| **Dep** | β_3_ / β_1_ | **Dep** | (β_3_ + β_12_) / (β_1_ + β_10_) |
| **Age** | β_4_ / β_1_ | **Age** | (β_4_ + β_13_) / (β_1_ + β_10_) |
| **dis1** | β_5_ / β_1_ | **dis1** | (β_5_ + β_14_) / (β_1_ + β_10_) |
| **dis2** | β_6_ / β_1_ | **dis2** | (β_6_ + β_15_) / (β_1_ + β_10_) |
| **ill1** | β_7_ / β_1_ | **ill1** | (β_7_ + β_16_) / (β_1_ + β_10_) |
| **ill2** | β_8_ / β_1_ | **ill2** | (β_8_ + β_17_) / (β_1_ + β_10_) |
| **Model 1**  **Variable** | **Donor MRS** | **Model 1**  **Variable** | **Healthcare professional MRS** |
| **Wait** |  | **Wait** |  |
| **Tiss** | (β_2_ + β_20_) / (β_1_ + β_19_) | **Tiss** | (β_2_ + β_29_) / (β_1_ + β_28_) |
| **Dep** | (β_3_ + β_21_) / (β_1_ + β_19_) | **Dep** | (β_3_ + β_30_) / (β_1_ + β_28_) |
| **Age** | (β_4_ + β_22_) / (β_1_ + β_19_) | **Age** | (β_4_ + β_31_) / (β_1_ + β_28_) |
| **dis1** | (β_5_ + β_23_) / (β_1_ + β_19_) | **dis1** | (β_5_ + β_32_) / (β_1_ + β_28_) |
| **dis2** | (β_6_ + β_24_) / (β_1_ + β_19_) | **dis2** | (β_6_ + β_33_) / (β_1_ + β_28_) |
| **ill1** | (β_7_ + β_25_) / (β_1_ + β_19_) | **ill1** | (β_7_ + β_34_) / (β_1_ + β_28_) |
| **ill2** | (β_8_ + β_26_) / (β_1_ + β_19_) | **ill2** | (β_8_ + β_35_) / (β_1_ + β_28_) |
| **Model 2**  **Variable** | **Non-ethnic minority MRS** | **Model 2**  **Variable** | **Ethnic minority MRS** |
| **Wait** |  | **Wait** |  |
| **Tiss** | β_2_ / β_1_ | **Tiss** | (β_2_ + β_11_) / (β_1_ + β_10_) |
| **Dep** | β_3_ / β_1_ | **Dep** | (β_3_ + β_12_) / (β_1_ + β_10_) |
| **Age** | β_4_ / β_1_ | **Age** | (β_4_ + β_13_) / (β_1_ + β_10_) |
| **dis1** | β_5_ / β_1_ | **dis1** | (β_5_ + β_14_) / (β_1_ + β_10_) |
| **dis2** | β_6_ / β_1_ | **dis2** | (β_6_ + β_15_) / (β_1_ + β_10_) |
| **ill1** | β_7_ / β_1_ | **ill1** | (β_7_ + β_16_) / (β_1_ + β_10_) |
| **ill2** | β_8_ / β_1_ | **ill2** | (β_8_ + β_17_) / (β_1_ + β_10_) |

Thus to establish (model 2) whether preferences for tissue matching differed between ethnic and non-ethnic minorities, the hypothesis is preferences for tissue match MRS for non-ethnic and ethnic minorities identical (p ≤ 0.05 indicates a difference at the 5% level). This is tested using a Wald test of the following restriction β_2_ / β_1_ = (β_2_ + β_11_) / (β_1_ + β_10_).
